# Short Telomeres in Key Tissues Initiate Local and Systemic Aging in Zebrafish

**DOI:** 10.1371/journal.pgen.1005798

**Published:** 2016-01-20

**Authors:** Madalena C. Carneiro, Catarina M. Henriques, Joana Nabais, Tânia Ferreira, Tânia Carvalho, Miguel Godinho Ferreira

**Affiliations:** Instituto Gulbenkian de Ciência, Oeiras, Portugal; University of Montreal/IRIC, CANADA

## Abstract

Telomeres shorten with each cell division and telomere dysfunction is a recognized hallmark of aging. Tissue proliferation is expected to dictate the rate at which telomeres shorten. We set out to test whether proliferative tissues age faster than non-proliferative due to telomere shortening during zebrafish aging. We performed a prospective study linking telomere length to tissue pathology and disease. Contrary to expectations, we show that telomeres shorten to critical lengths only in specific tissues and independently of their proliferation rate. Short telomeres accumulate in the gut but not in other highly proliferative tissues such as the blood and gonads. Notably, the muscle, a low proliferative tissue, accumulates short telomeres and DNA damage at the same rate as the gut. Together, our work shows that telomere shortening and DNA damage in key tissues triggers not only local dysfunction but also anticipates the onset of age-associated diseases in other tissues, including cancer.

## Author Summary

Why, and how, organisms age and ultimately die is a key question of modern biology. Telomeres are considered molecular timekeepers determining cellular lifespans. Within an organism, tissue proliferation is expected to dictate the rate at which telomeres shorten. Using zebrafish, an organism with human-like telomeres, we set out to test whether, in natural aging, proliferative tissues age faster than non-proliferative in a telomere-dependent manner. We found that telomeres shorten with age to a level where they trigger telomere associated DNA damage and culminate in tissue dysfunction, independently of high or low tissue proliferation rates. Specifically, short telomeres accumulate in the gut, a highly proliferative tissue, and in the muscle, a low proliferative tissue, working as direct predictors of cellular damage prior to onset of intestinal inflammation and myocyte degeneration. Based on our data, we propose a model where telomere shortening in these key tissues is sufficient to trigger damage in others and precedes the onset of organism age-associated diseases, namely cancer. Thus, tissue-specific telomere length is limiting for local and systemic physiological integrity, leading to tissue degeneration and disease in aging.

## Introduction

Telomeres are structures composed of DNA repeats and a specific set of proteins (the shelterin complex) that protect the ends of eukaryotic chromosomes. Telomeres have two main functions: 1) to prevent recognition of chromosome-ends as deleterious DNA double strand breaks (DSBs) and 2) to ensure complete DNA replication by recruitment of telomerase, a reverse transcriptase that that synthesizes new telomeric DNA repeats [1,2]. Due to the “end-replication problem”, telomeres shorten with each round of cell division in cells that do not express telomerase [3,4]. On average, human cells lose 50–100 bp from their extremities in every round of division [5–7].

Telomerase expression is restricted in most human somatic cells [8]. Consequently, as observed in population studies, telomeres shorten significantly during human aging [9,10]. The rate of telomere decline is most pronounced in early life, from birth to puberty, slowing down in adulthood [9,11]. Telomere erosion is a strong barrier to cell proliferation and thus thought to constitute a tumor suppression mechanism [12]. However, by limiting the function of stem cell reserves necessary for tissue renewal, telomere shortening has also been proposed to be a strong promoter of aging-associated degenerative phenotypes [10,13,14]. Indeed, short telomeres are a frequent feature of diseases that anticipate facets of aging, such as Dyskeratosis Congenita, DC [15–17]. DC patients harbor mutations in several components involved in telomere maintenance such as DKC1 (dyskerin), a component of the active telomerase enzyme complex [18], hTR (telomerase RNA), hTERT (telomerase reverse transcriptase) and in shelterin component TINF2 [19–22]. Mutations in DKC1 associate specifically with an X-linked form of DC [23]. Short telomeres as cause of age-related disease is further supported by other syndromes, such as Idiopathic Pulmonary Fibrosis (IPF) and Hoyeraal-Hreiderasson [19,24]. These “telomeropathies” exhibit a pattern of genetic anticipation, in which later generations suffer from increased disease severity [15,25,26]. These diseases are characterized by dysfunction of proliferative tissues, namely bone marrow, lung and liver, ultimately leading to multi-organ failure. Dysfunction and disease severity can be predicted by the extent of short telomere accumulation in different tissues [27,28]. Apart from these symptoms, DC patients show severe mucocutaneous alterations and an increased susceptibility for developing cancer [15].

A direct association between telomere shortening and tissue specific dysfunction in vertebrates has been clearly demonstrated in late-generation telomerase knockout mice (both catalytic enzyme, mTERT-/- and RNA subunit, mTR-/-) and, more recently, in telomerase deficient zebrafish [29–33]. Telomerase plays a pivotal role in high-turnover mouse and zebrafish tissues [29,30,32]. In G6 mTR-/- mice, intestinal epithelial cells and male germ cells have reduced proliferative capacity and increased apoptosis, resulting in tissue atrophy and infertility [32]. In low proliferative tissues, such as heart and liver, G4 mTERT-/- mice incur metabolic failure by repressing master regulators of mitochondrial biogenesis, via upregulation of p53 [34]. In zebrafish, first generation telomerase mutants show a tissue and time dependent dysfunction that associates with short telomeres and diminished proliferative rates [30].

Telomere shortening can also disrupt tissue integrity in a non-cell autonomous manner, by inducing cell senescence. Senescent cells accumulate with age in a variety of mammalian tissues, such as skin and eye [35]. These cells secrete a specific set of molecules, including inflammatory cytokines and chemokines, growth and survival factors, which are referred to as the “Senescence-Associated Secretory Phenotype”, SASP [36]. SASP creates a microenvironment within and between tissues which compromises homeostasis and promotes features of tumor malignancy, including invasiveness [36,37].

Further evidence supports extratelomeric functions for telomerase (which are independent of telomere elongation) and that may influence aging phenotypes. Telomerase stimulates (stem) cell proliferation by acting as a cofactor of the beta-catenin transcriptional complex [38], and promotes tumor development by potentially activating Myc and Wnt signaling [39,40]. Additional non-canonical roles of telomerase involve its interaction with the RNA component of mitochondrial RNA processing endoribonuclease (RMRP), which produces double stranded RNAs that can be processed into siRNAs [41]. Finally, telomerase can be targeted to the mitochondria affecting its function, superoxide production and apoptosis [42,43].

While short telomeres may underlie part of the cascade of tissue homeostasis decline with time, aging mammalian species are affected by many other factors, such as mitochondrial dysfunction, epigenetic alterations and impaired proteostasis [44–46]. It remains to be defined in which tissues telomeres shorten to critical levels with natural aging, triggering DNA Damage Responses (DDR) and, ultimately, organ dysfunction. Identifying the tissues where short telomeres dictate phenotypes of aging and how these tissues then influence the organism as a whole is crucial for understanding the impact of telomere length on aging.

Zebrafish has emerged as an important vertebrate model to study both telomere biology and aging [29,30,47–50]. First generation telomerase mutant zebrafish age and die prematurely [29,30], reminiscent of human telomere shortening syndromes. Yet, a detailed analysis of organ dysfunction and respective telomere shortening dynamics throughout lifetime in wild type animals remains largely unexplored. We conducted a comparative prospective study of telomere dynamics, DNA Damage Response (DDR) and aging-related dysfunction and disease in different tissues in both WT (from 3 to 42 months) and *tert*^*-/-*^ zebrafish (from 3 to 12 months, when at least 50% of *tert*^*-/-*^ mutants are dead). This allowed us to define the choreography (timings and kinetics) of age-related lesions and how these are conserved between WT and *tert*^*-/-*^. We discovered that *tert*^*-/-*^ mutants anticipate the tissue-specific dysfunctional events observed during normal zebrafish aging, including tumorigenesis.

## Results

### Zebrafish telomere length varies between different tissues

**Fig 1:**
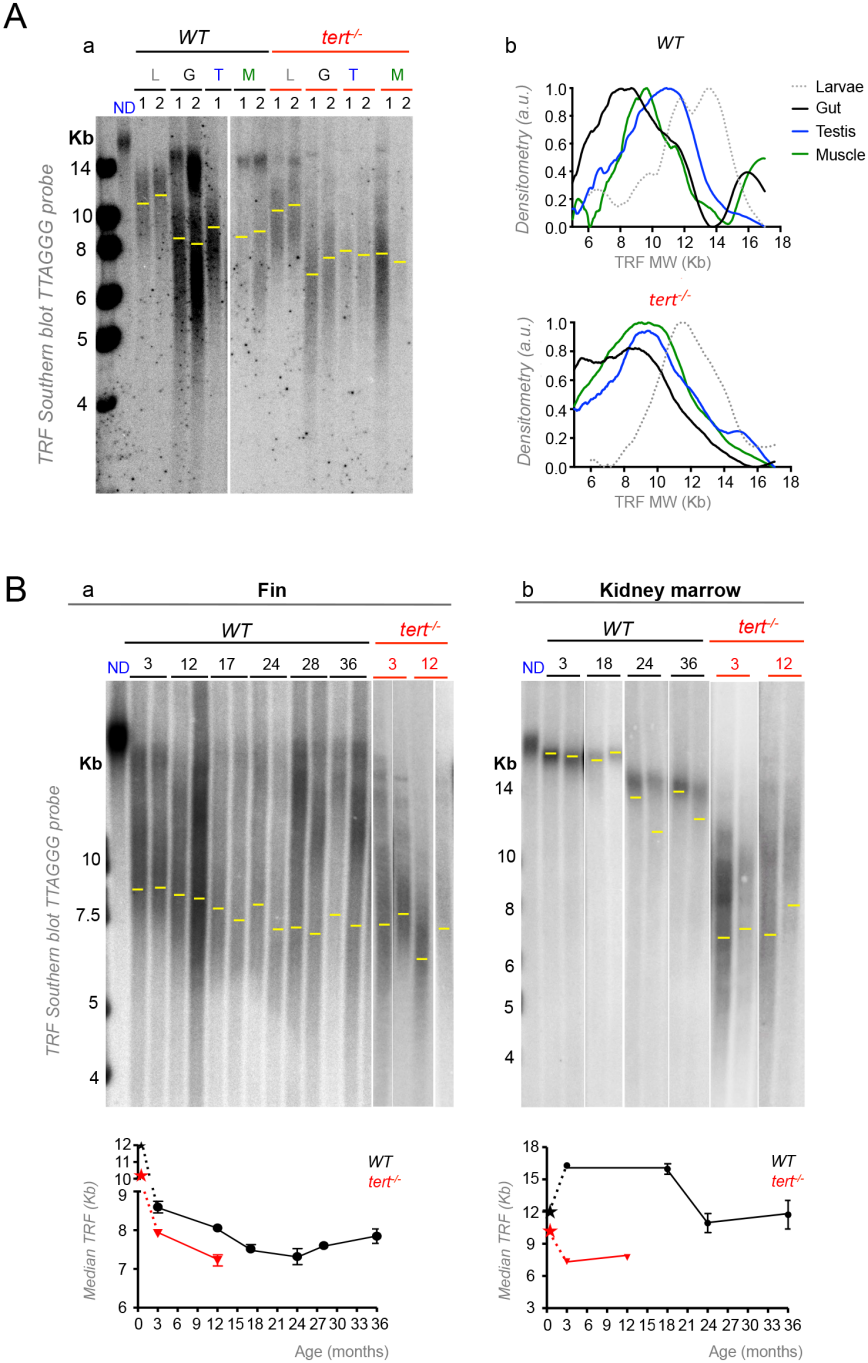
Different zebrafish tissues have different telomere lengths and follow specific dynamics of shortening with aging. A) Representative images of telomere restriction fragment (TRF) analysis of genomic DNA by Southern Blot (random primer-labelled telomeric probe (TTAGGG)n ^32^P-dCTP). Aa) WT telomeres are longer in whole larvae (“L”, ca. 12 kb) and shorter in 3 month-old zebrafish gut (G), testis (T) and muscle (M), but show significant variation in length between tissues (shown for two independent WT zebrafish “1” and “2” in Fig 1Aa, except for testis where only zebrafish “1” is shown; densitometry shown for zebrafish “1”, Fig 1Ab). WT have longer telomeres in testis (ca.9.9 Kb), followed by muscle (ca. 9.4 Kb) and, finally, gut (8.4 Kb). These differences are globally maintained in *tert*^*-/-*^ tissues (ca. 8.3 Kb in the testis, ca. 8.5 in the muscle and ca. 7.8 Kb in the gut; shown for two 3-month old zebrafish “1” and “2” in Fig 1Aa; densitometry shown for individual “1”, Fig 1Ab). Yellow line indicates median telomere length, mTL, for each tissue sample/lane. B) WT telomeres significantly shorten in the fin and kidney marrow with age, but follow different shortening kinetics with time (black star represents WT larvae telomeres; two zebrafish shown for each age after sexual maturation– 3, 12, 17, 24, 28, 36 months for the fin and 3, 18, 24, 36 months for the kidney marrow). Ba) The telomere shortening rate within the same individual (measured by cutting different fins in different time points) is of 45 bp/month and 90 bp/month for two independent zebrafish (in Fig Ba, lanes 1 and 2 of each time point are the same individual over time between the ages of 3 and 24 months). WT telomeres in the fin match the shorter length of 12-month old *tert*^*-/-*^ mutants by 18–24 months (N = 4 for WT and N = 3 for *tert*^*-/-*^ fin; red star represents *tert*^*-/-*^ larvae telomeres; Fig 1Ba), but WT kidney marrow telomeres never reach *tert*^*-/-*^ levels (N = 3–6 per time point for adult WT and N = 3 for adult *tert*^*-/-*^ mutants, Fig 1Bb). TRF mean sizes were calculated as previously described [54]. mTL data is represented as mean +/- SEM.

Human population studies revealed variation in telomere length between different tissues, irrespective of their proliferation rates [51]. In birds, variation in length was also found between different somatic tissues [52]. However, population averages are unable to discriminate if telomere shortening precedes tissue dysfunction in aging. Zebrafish has telomeres of human like length, ca. 5–16 kb [30,47,53] and absence of telomerase limits its lifespan [29,30]. To test if different zebrafish tissues have different telomere lengths within an individual, we dissected juvenile zebrafish and performed Telomere Restriction Fragment (TRF) [54] analysis of high proliferative (gut, testis) and low proliferative tissues (muscle). We observed that, whereas at larval stages telomeres are more homogeneous and longer (larvae, “L”, Fig 1Aa), different tissues have acquired different telomere lengths by the time of sexual maturity, at 3 months (Fig 1Ab). We observed most WT tissues have a mixture of long and short populations of TRFs and *tert*^*-/-*^ mutants predominantly have the latter (Fig 1Ab). The longer telomere population is of the same size observed in blood and thus likely to reflect circulating cells in all tissue samples. We therefore decided to use the median Telomere Length (mTL) as a conservative measurement of telomeres in different cell populations/tissues.

Three-week-old zebrafish larvae had long telomeres (ca.12 Kb) that declined with growth and development (Fig 1Aa). Sexually mature males had long mTL in the testis (ca. 9.9 Kb) followed by the muscle (ca. 9.4 Kb) while the shortest telomeres measured were in the gut (ca. 8.4 Kb, Fig 1Aa). Strikingly, the relative telomere length difference between highly proliferative tissues testis and gut are globally maintained in *tert*^*-/-*^ mutant tissues (*tert*^*-/-*^ larvae had telomeres of ca. 11.6 Kb which declined to ca. 8.3 Kb in the testis and gut ca. 7.8 Kb; Fig 1Aa). This suggests that, like in humans, where telomerase activity is restricted in most tissues, telomere length and set points are established during zebrafish development.

### Zebrafish telomeres decline with age and the rate of shortening varies between tissues

In order to determine the dynamics of telomere shortening with aging in different tissues, we performed TRF analysis at different ages in the fin, a less proliferative tissue, which we previously reported to have shorter telomeres, and in the kidney marrow (the hematopoietic organ in zebrafish), a proliferative tissue with long telomeres [30]. We measured telomeres in four individuals and observed significant shortening of WT fin telomere length with age at the population level (Fig 1Ba). We detected a linear decline on mTL from 3 to 24 months of age, ranging ca. 8.6 Kb to 7.3 Kb (Fig 1Ba). From 24 months of age onwards, we did not detect further telomere shortening (Fig 1Ba), suggestive of an exhaustion of proliferation potential in later life stages. By taking advantage of the well-studied regenerative capacity of zebrafish fin, we were able to calculate the telomere shortening rate for two zebrafish individuals for their first 24 months of life, by taking advantage of the well-studied regenerative capacity of zebrafish fin. We estimated a decline of 45 bp/month and 90 bp/month respectively. Since telomerase expression was reported to increase during fin regeneration [47], we performed this experiment by sampling telomere length in the different fins for each individual at different ages.

Contrary to any other tissue analyzed, WT kidney marrow telomeres dramatically elongated from larvae to fully developed adult stages (S1 Fig) and shortened with age after 18 months (Fig 1Bb). At the population level, by averaging different individuals’ telomere median length (N = 3–6 for each time point from 3 months onwards), no appreciable shortening could be detected in the first 18 months of life, after which telomeres declined (Fig 1Bb). This is suggestive of higher telomerase activity and, consequently, lesser telomere shortening in kidney marrow than in the fin.

Telomeres shortened significantly with age in the fin of WT, matching the length of 12-month old *tert*^*-/-*^ mutants (N = 3 for each time point) by 18–24 months (Fig 1Ba). In contrast, WT telomeres of the kidney marrow never reached the length of *tert*^*-/-*^ mutants (N = 3 for each time point) during their lifetimes (Fig 1Bb). These data show that telomere median length, as measured by TRF, correlates directly with zebrafish age up to 18–24 months albeit in a tissue specific manner. In addition, mTL shortening reaches a limit bellow which we are unable to detect further decrease. As previously seen by others [9,11], this is likely due to a selection process that eliminates cells with extremely short telomeres thus creating a virtual barrier to telomere decrease.

### WT gut and muscle telomere lengths reach *tert*^*-/-*^ mutants’ levels with aging

We have previously shown that *tert*^*-/-*^ mutants have shorter telomeres than WT siblings already by 3 months (Fig 1A and [30]). As consequence, *tert*^*-/-*^ mutants develop premature cell proliferation defects and DDR which culminate in early tissue degeneration [30]. Thus, in our study, we used *tert*^*-/-*^ mTL as proxy for the presence of critically short telomeres that cause tissue dysfunction in WT.

**Fig 2:**
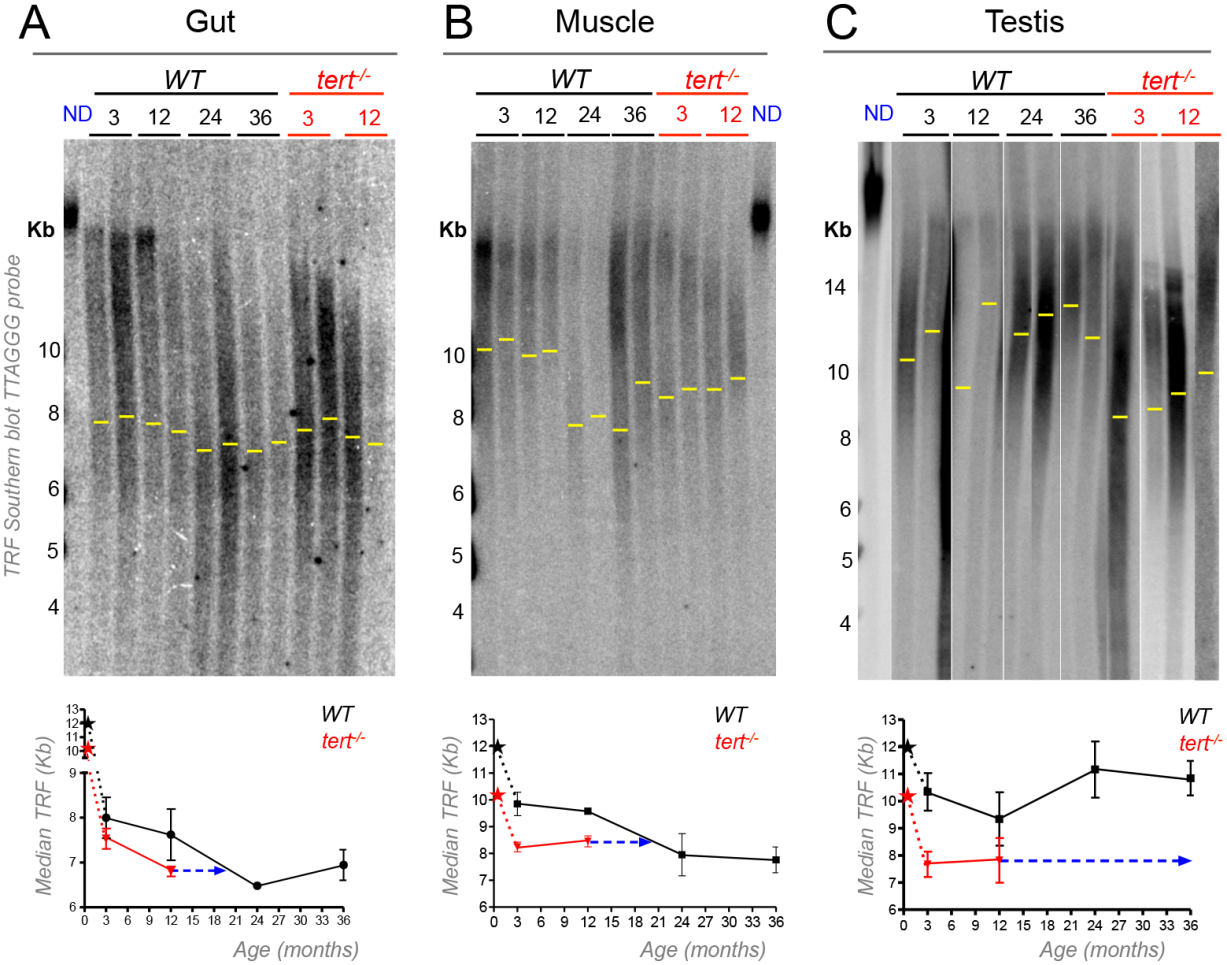
Telomeres shorten in WT gut and muscle, but not in testis, with aging reaching the length of *tert*^*-/-*^ tissues. A-C) Representative images of telomere restriction fragment (TRF) analysis of genomic DNA by Southern Blot (random primer-labelled telomeric probe (TTAGGG)n ^32^P-dCTP) and quantifications of median telomere length. Black and red stars represent WT and *tert*^*-/-*^ larvae telomeres, respectively, in quantifications of median TRF over time. WT telomeres shorten linearly with aging from 3 to 24 months, in A) gut (N = 3–4 per time point) and B) muscle (N = 3–4 per time point), stabilizing in later ages. C) No significant telomere shortening is detected in the testis (N = 5–6 per time point). Around 20 months of age WT telomeres reach the shorter length of 12 month-old *tert*^*-/-*^ in the gut (graph in fig A–ca. 6.8 Kb) and muscle (graph in fig B–ca. 8.5 Kb) but not in testis (graph in fig C), indicated by blue arrow. N = 3–4 per time point for *tert*^*-/-*^ gut and muscle, N = 4–6 per time point for *tert*^*-/-*^ testis. TRF mean sizes were calculated as previously described [54]. Data are represented as mean +/- SEM.

To probe for accumulation of short telomeres, we determined the variation in telomere length in WT proliferative tissues gut and testis and less proliferative tissue muscle, over time (N = 3–4 per time point for gut and muscle; N = 5–6 per time point for testis; Fig 2). While WT kidney marrow mTL shortened within a range of ca. 16–11Kb (Fig 1Bb), other tissues’ mTL shortened to lower ranges of 11–6Kb (Fig 2, densitometries shown for one individual per age in S2 Fig). For the time points tested after sexual maturity, mTL ranged between ca. 8.0–6.5 Kb in the gut, ca. 10.2–7.8 Kb in the muscle and ca. 11.1–9.3 Kb in the testis (Fig 2A–2C). We further detected significant mTL shortening in the gut and muscle of WT at 24 months (Fig 2A and 2B). Similar to what was observed in the fin, gut and muscle telomeres declined significantly in a linear fashion between 3 and 24 months in WT, stabilizing in the last time point of 36 months (Fig 2A and 2B). Because the muscle is a low proliferative tissue, telomere shortening with age is likely to rely on factors that are independent of local cell replication. Apart from cell division, reactive oxygen species (ROS) are prime candidates for generating damage at telomeres and, consequently, promoting telomere shortening [55]. We tested if the levels of ROS increased differently with aging in tissues with distinct turnovers, by measuring total ROS in the gut and muscle of 6 and 36 month-old WT zebrafish, using the cell-permeant 5- chloromethyl-2’,7’-dichlorodihydrofluorescein diacetate (CM-H2DCFDA). We found that the muscle accumulated significantly (ca. 12x) higher levels of ROS with aging (p = 0.04, S3 Fig). In contrast, we could not find significant differences in ROS levels between the guts of 6 and 36 month-old zebrafish (S3 Fig).

*tert*^*-/-*^ mutants had shorter mTL already by 3 months of age in all tissues and we could not detect further significant shortening by the age of 12 months (N = 3–4 per time point for gut and muscle, N = 4–6 per time point for testis; Fig 2A–2C). When comparing WT and *tert*^*-/-*^, we found that telomeres in both gut and muscle of old WT reached the length of 12 month old *tert*^*-/-*^ (6.8 Kb for gut and 8.5 Kb for muscle; Fig 2A and 2B). We estimated that short telomeres in WT reached the level of *tert*^*-/-*^ mutants around the age of 20 months (indicated by blue arrow in Fig 2A and 2B). In contrast, WT telomeres did not shorten appreciably with age in the testis (indicated by blue arrow in Fig 2C). In summary, telomeres shorten in old WT zebrafish to the level of *tert*^*-/-*^ mutants the fin, gut and muscle, but not in testis and kidney marrow, regardless of their distinct proliferation rates.

### Short telomeres precede activation of DDRs

**Fig 3:**
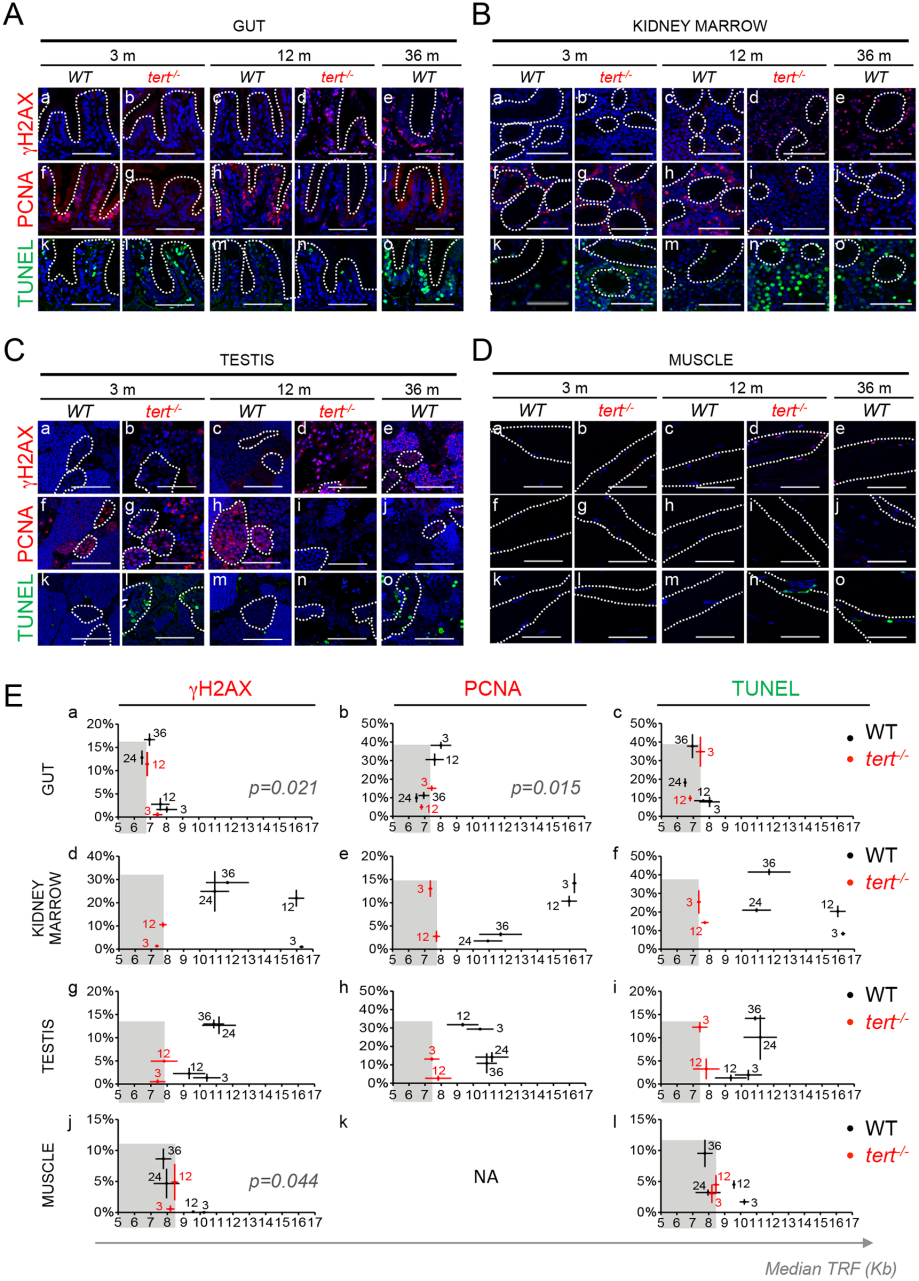
Shortening of mTL anticipates accumulation of DDR markers, decrease in cell proliferation and increase in apoptotis with age. A-D) Representative immunofluorescence images of DNA damage (γH2AX), proliferation (PCNA) and apoptosis (TUNEL) for gut (A—dashed outline identifies the villi), kidney marrow (B), testis (C) and muscle (D—dashed outline identifies the fibers) of WT (at 3, 12 and 36 months) and *tert*^*-/-*^ mutant siblings (at 3 and 12 months). All tissues show a significant increase in cells bearing five or more γH2AX foci by 12 months of age in *tert*^-/-^ zebrafish (panels b, d) and by 36 months in WT zebrafish (panels a, c and e). Increased DNA Damage Response (DDR) correlates with shorter mTL in Ea) Gut (p = 0.021) and Ej) Muscle (p = 0.044) but not Ed) Kidney Marrow or Eg) Testis. Grey shaded area identifies the median telomeric length at which significant DDR activation is observed in *tert*^*-/-*^ mutants (Ea, Ed, Eg, Ej). Proliferative tissues, A) gut B) kidney marrow and C) testis, show a sustained decrease in cell proliferation by 12 months of age in *tert*^*-/-*^ (panels g, i) and by 36 months in WT tissues (panels f, h and j). Eb) Decrease in proliferation correlates with shorter mTL in the gut (p = 0.015). Grey shaded area identifies the median telomeric length at which significant proliferation defects are observed in *tert*^*-/-*^ mutants (Eb, Ee, Eh). *tert*^*-/-*^ mutants’ A) gut B) kidney marrow and C) testis show increased apoptotic responses at 3 months when compared with WT controls (panel l). WT zebrafish show a continuous accumulation of apoptotic signals with age (panels k, m, o). Apoptotic responses are not anticipated by shorter mTL (Ec, Ef, Ei, El). Grey shaded area identifies the median telomeric length at which significant apoptotic responses are observed in *tert*^*-/-*^ mutants (Ec, Ef, Ei, El). Most DDRs and apoptotic signals locate to the proliferative zone of maturing spermatocytes (C—dashed outline). WT and *tert*^*-/-*^ age groups are indicated in each graph by black and red colored numbers, respectively. Immunofluorescence (IF) quantifications were performed in at least 3 different fields of view for 3–5 different individuals per time point per genotype. Gut IF quantifications were calculated as number of positive cells per “crypt” zone. Other tissues' IF was quantified as overall % positive cells. Scale bar = 50 μm. N = 3–6 for tissue mTL quantifications per genotype per time point (x-axis in graphs of Fig 3E). IF and mTL quantifications are represented as mean +/- SEM.

One of the hallmarks of aging is the general accumulation of DNA damage [56]. This leads to robust activation of DDR pathways, which accumulate preferentially at short or damaged telomeres [57,58]. This process is thought to underlie the establishment of cellular senescence. In order to determine if short telomeres precede activation of persistent DDR in tissues, we measured the appearance of nuclear foci of phosphorylated H2AX at Ser139 (γ-H2AX) over time. Presence of short telomeres in *tert*^*-/-*^ tissues resulted in the accumulation of DDR markers. From 3 to 12 months, DDR markers increased significantly from 0.5 to 11.4% in the gut (p = 0.0077, Fig 3Ab, 3Ad and 3Ea; S4 Fig), 1.4 to 10.5% in the kidney marrow (p = 0.0096, Fig 3Bb, 3Bd and 3Ed; S4 Fig) and 0.5 to 5.0% in the testis (p = 0.0065, Fig 3Cb, 3Cd and 3Eg; S4 Fig) and showed a slight tendency to increase in the muscle (p = 0.35, Fig 3Db, 3Dd and 3Ej; S4 Fig). During this period, telomere length was always shorter in *tert*^*-/-*^ tissues when compared with WT lengths, ranging ca. 7.4–6.8 Kb in the gut, ca. 7.3–7.7 Kb in the kidney marrow, ca. 7.4–7.8 Kb in testis and ca. 8.2–8.5 Kb in muscle (Figs 1 and 2).

In order to investigate whether short telomeres dictated the onset of tissue dysfunction in WT tissues, we asked if changes in molecular markers related to DDR, cell proliferation and apoptosis were correlated with mTL decline (Fig 3E). In gut and muscle of old WT, shorter mTL directly correlated with the increase in DDR markers, which reached *tert*^*-/-*^ levels (p = 0.021 and p = 0.044, Fig 3Ea and 3Ej). More specifically, the percentage of WT cells bearing five or more γH2AX foci increased from 2% to 18% in the gut (p<0.0001) and from 0 to 9% in muscle (p = 0.0007), between the ages of 3 and 36 months, as mTL declined from 8 to 6.9Kb and from 10.2 to 7.8Kb respectively (Fig 3Aa–3Ae, 3Da–3De, 3Ea and 3Ej). However, an increase in DDR with age, from 3 to 36 months, was also detected in the testis and kidney marrow (of 12%, p = 0.0027, and 28%, p = 0.0003, respectively) of WT zebrafish, even though these tissues failed to significantly shorten their telomeres to *tert*^*-/-*^ levels (Fig 3Ba–3Be and 3Ca–3Ce, 3Ed and 3Eg). Thus, DDR activation in cells of naturally aged zebrafish strongly correlates with telomere shortening specifically in the gut and muscle. However, some highly proliferative tissues, such as testis, exhibit marks of DDR independently of critically short telomeres, as set by *tert*^*-/-*^ mTL. To determine what is the contribution of short-telomere induced damage to the increase in DDR in aging, we performed telomere- Fluorescence In Situ Hybridization (FISH) in γH2AX immuno-stained cells isolated from WT and *tert*^*-/-*^ gut (at 3, 12 and 36 months for WT and at 3 months for *tert*^*-/-*^, N = 3 per age/genotype). The number of three of more telomere induced foci (TIF) increased significantly from 3 to 36 months in WT gut (p = 0.0001, arbitrary line indicates 3 or more TIFs, S5A and S5B Fig). We also found a significant accumulation in TIFs in 3 month-old *tert*^*-/-*^ gut, when comparing with age-matched 3-month old WT controls (p = 0.0001, arbitrary line indicates 3 or more TIFs, S5A and S5B Fig). Our data demonstrates that the presence of dysfunctional telomeres is an important contributor to the age-associated increase in DDR in the zebrafish gut.

We next asked whether the observed decline in mTL could anticipate other cellular phenotypes of aging. We looked for changes in rates of cell proliferation and apoptosis emerging with time and correlated them with mTL decline. Indeed, using PCNA immunostaining as readout for cell division, we observed decreased proliferation in the gut and testis of *tert*^*-/-*^ mutants already by 3 months of age and by 12 months in the kidney marrow (Fig 3A–3C). Accordingly, the percentage of proliferating cells dropped in the gut of older WT individuals (3 month vs. 36 months) from 38% to 10% (p<0.0001, Fig 3Af–3Aj and S4 Fig) and this strongly correlated with telomere shortening (p = 0.015, Fig 3Eb). Testis and kidney marrow also showed a marked decrease in cell division with aging. We observed a decline of 19% (p = 0.0005) and 12% (p< 0.0001) of PCNA stained cells between 3 months and 36 months, in testis and kidney marrow respectively (Fig 3Bf–3Bj, 3Cf–3Cj and S4 Fig). Overall alterations in proliferation were particularly evident in areas where stem cells and progenitors reside, such as the basal compartment of the seminiferous tubules in the testis and the base of intestinal villi (Fig 3Af–3Aj, 3Cf–3Cj). However, similarly to DDR, testis and kidney marrow showed decreased proliferation with aging regardless of appreciable telomere length variation (Fig 3Ee and 3Eh). In a similar way, decrease of mTL with age did not correlate with apoptotic index in any studied tissue (p = 0.31 for gut, p = 0.13 for muscle, p = 0.40 for testis, p = 0.32 for kidney marrow, Fig 3Ec, 3Ef, 3Ei and 3El). Nevertheless, a significant increase of apoptosis with age was observed by the higher TUNEL staining, detected in all 36 month-old WT tissues when compared with 3-month old groups (p = 0.031 for gut, Fig 3Ak–3Ao; p = 0.0008 for kidney marrow, Fig 3Bk–3Bo; p = 0.0091 for testis, Fig 3Ck–3Co; p = 0.0279 for muscle, Fig 3Dk–3Do and S4 Fig).

Senescent cells gradually accumulate in aging tissues [35] and critically short telomeres determine the onset of replicative senescence in human cells [59]. During aging of primates, the number of cells bearing markers of senescence and telomere dysfunction increases exponentially in the skin [60]. We assessed whether mTL decline would also correlate with the increase in senescence found in aging zebrafish tissues. Our results showed that short telomeres in the gut anticipated the increase in senescence measured by senescence-associated β-galactosidase (SA-β-gal) for aging WT and for *tert*^*-/-*^ mutants (p = 0.10, S6 Fig). As observed for DDR levels, even though senescence was visible in testis and kidney marrow, there was no correlation of mTL decline with increased senescence with age in these tissues (S6 Fig).

Thus, using *tert*^*-/-*^ mutant zebrafish mTL as reference, the above analysis establishes the gut and muscle as primary tissues where short telomeres anticipate decreased proliferation and increased DDR with aging.

### Accumulation of short telomeres and DDRs culminate in aging-associated tissue dysfunction

Short telomeres, as defined by *tert*^*-/-*^ length, accumulate with zebrafish aging and correlate with activation of persistent DDRs in specific tissues: gut and muscle. We therefore tested whether these cellular phenotypic alterations preceded a decline in tissue integrity. We first looked for changes in tissue inflammation. Aging is characterized by a systemic chronic pro-inflammatory condition with rising levels of TNF, IL1 and IL6 [61–64]. This “proinflammatory state” has been postulated to lead to several chronic degenerative disorders [61,62,65]. We found that *tert*^*-/-*^ zebrafish developed early (by 6 months) enteritis and epithelial erosion that was further aggravated in older individuals (18% incidence by 12 months, N = 12 in a total of 66 *tert*^*-/-*^ mutants analyzed; S7A Fig). As short telomeres accumulated in the gut, the incidence of enteritis increased from 0% to 13% in aged WT zebrafish (3 month vs. 36 months, N = 30 in a total of 238 zebrafish analyzed, S7A Fig).

**Fig 4:**
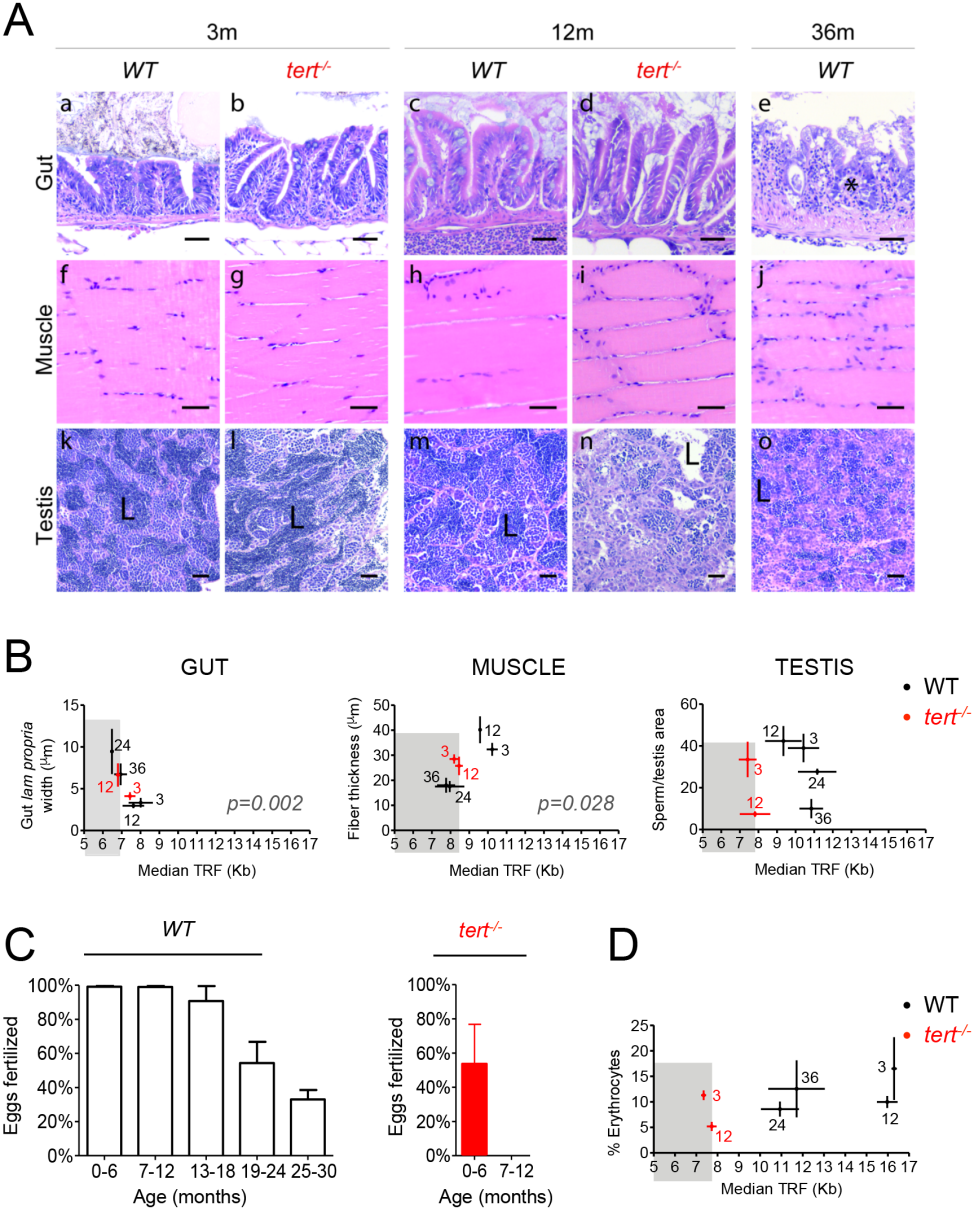
Telomere shortening culminates in tissue defects associated with aging. A) Representative haematoxylin and eosin-stained sections of gut, muscle and testis from WT and *tert*^*-/-*^ siblings. By 12 months of age, *tert*^*-/-*^ mutants (N = 3) show inflammatory cell infiltration of the *lamina propria* of the gut (panel b, d), myocyte atrophy and degeneration (characteristic of sarcopenia—panels i), and reduced numbers of mature spermatozoa (panels l, n). Similarly, by 36 months, WT show similar lesions in the gut (N = 5, panels e) and muscle (N = 5, panels j); and with aging, WT testis show a gradual decrease in the number of spermatozoa (panels k, m, o, N = 5). B) Among these age-related histological lesions, intestinal inflammation and sarcopenia correlate with shortening of mTL (p = 0.002 and p = 0.028, respectively), while no correlation is found for reduction in mature spermatozoa numbers. Grey shaded area identifies the median telomeric length at which significant intestinal inflammation, sarcopenia and defects in production of mature spermatozoa are observed in *tert*^*-/-*^ mutants. WT and *tert*^*-/-*^ age groups are indicated in each graph by black and red colored numbers, respectively. C) In accordance with lower levels of spermatozoa production at later ages, WT show a decreased ability to successfully fertilize female eggs from 24 months of age onwards (N = 4). *tert*^*-/-*^ have impairment of reproductive ability by 6 months and complete lack of function by 12 months (N = 3). D) Erythrocyte levels (N = 5) decrease with aging in WT and *tert*^*-/-*^ mutants’ kidney marrow, indicative of anemia, but this is not predicted by mTL decline in this tissue. Grey shaded area identifies the median telomeric length at which a significant decrease in erythrocyte levels is observed in *tert*^*-/-*^ mutants. Scale bar = 50 μm. N = 3–6 for tissue mTL quantifications per genotype per time point (x-axis in graphs of Fig 4B and 4D). Data are represented as mean +/- SEM.

We also looked for general alterations in intestinal structure. The gastrointestinal tract is known to suffer several alterations in microbiota composition, mucosal immune system and regeneration capacities with aging [66–68]. These changes compromise normal organ function and nutrient absorption. We detected a progressive tendency for thickening of the *lamina propria* and submucosa in the anterior part of the intestine with *tert*^*-/-*^ mutants’ aging, from 3 to 12 months, associated with inflammatory cell infiltration (Fig 4Ab and 4Ad, quantified in 4B). WT zebrafish showed significant thickening of the *lamina propria* and submucosal layers at 36 months (Fig 4Ae, indicated by asterisk, quantified in Fig 4B). This morphological feature correlated well with mTL decline in the gut (p = 0.002, N = 3 per time point, Fig 4B). However, we were unable to find significant changes in general gut villi length in older WT individuals that would correlate with shorter mTL (S7B Fig).

Parallel to the gut, decline in telomere length and activation of DDRs in the muscle prompted us to assess whether these could underlie alterations occurring in myocytes with age. Humans are known to lose muscle mass (also known as sarcopenia) progressively from adulthood to older ages primarily due to nutrient absorption defects (1–2% decrease per year after the age of 50, [69]). Atrophy of type IIa muscle fibers leads to a decay in muscle strength and motor capacity [69]. 12 month-old *tert*^*-/-*^ mutants showed significant muscle fiber thinning (when compared with 12 month WT siblings, Fig 4Ah–4Ai and 4B). We observed a 44% reduction in myocyte width in WT zebrafish by the age of 36 months, when compared with 3 months (p = 0.0001, Fig 4Af–4Aj), compatible with progressive sarcopenia. Severe myocyte degeneration and endomysisal edema (Fig 4Ai–4Aj) directly correlated with the presence of short telomeres (<8.5Kb, p = 0.028, Fig 4B). These results show that short telomeres directly correlate with the degenerative changes associated with aging, particularly intestinal inflammation and sarcopenia.

### Telomere shortening does not predict infertility or anemia associated with aging

Even though mTL does not appreciably decline to levels that compromise tissue integrity in testis and kidney marrow (when taking *tert*^*-/-*^ mTL as reference), we investigated how aging affected tissue homeostasis and function in these organs. Adult *tert*^*-/-*^ mutants showed reduced germ cell compartment by 12 months of age (Fig 4Ak–4An). Here, we show that WT male zebrafish display a progressive inability to produce mature spermatozoa with age (Fig 4Ak–4Ao, L- mature spermatozoa in the lumen of seminiferous tubules). In addition, 3% of WT zebrafish after 24 months of age (N = 7 in a total of 238 zebrafish analyzed, S7C Fig) displayed hyperplasia of seminiferous epithelium, mostly spermatogonia. Hyperplasia of spermatogonia in *tert*^*-/-*^ populations was residual (N = 1 in a total of 66 zebrafish analyzed). These results had not been described before and are consistent with telomerase being essential for expansion of the germ cell compartment in late generation telomerase knockout mice [32].

Mice harboring artificially shortened telomeres display defective spermatogenesis/cell proliferation in testis, failing to produce viable offspring [32,70,71]. Interestingly, even though we observed a marked decline in percentage of mature spermatozoa with age, this did not correlate with the presence of short telomeres in older WT zebrafish (Fig 4B). Decreased spermatozoa production levels resulted in decline in male fertility (Fig 4C). *tert*^*-/-*^ mutants showed a compromised ability (~50%) to fertilize female eggs at 6 months and became completely infertile by 12 months of age (Fig 4C). WT male zebrafish fertility peaked from 6 to 18 months of age and progressively decreased afterwards (~50% and ~30% of fertilization capacity by 24 and 30 months, respectively; Fig 4C, p< 0.0001). Interestingly, WT zebrafish decreased their ability to stimulate female spawning with age, from 24 months onwards, whereas these defects were already visible in 6-month-old *tert*^*-/-*^ males (S8A Fig). Fertility and egg spawning behavior were mildly but significantly affected with age in WT or *tert*^*-/-*^ mutant females (p = 0.0231 for WT females and p = 0.0357 for *tert*^*-/-*^ females, S8B and S8C Fig). Thus, although *tert*^*-/-*^ mutants anticipate the decline in spermatozoa production and fertility observed in older WT, the development of these phenotypes in a natural aging setting does not seem to depend on the presence of short telomeres in the gonads.

We observed that telomeres shortened later in life in the kidney marrow, but this shortening never matched the length of *tert*^*-/-*^ mutants (Fig 1B). We tested if, as in testis, *tert*^*-/-*^ mutants would recapitulate kidney marrow defects associated with aging. WT zebrafish showed lower levels of red blood cells (RBC) with aging (erythrocytes declined from 17% at 3 months to 6% at 24 months, p = 0.0002, Fig 4D and S9A Fig) and also a tendency for lower lymphocyte numbers (30% at 3 months vs. 10% at 33 months, p = 0.0011, S9A–S9C Fig). Conversely, the proportion of myeloid to lymphoid cells gradually increased with age (2% at 3 months versus 9% at 33 months, p<0.0001, S9A and S9C Fig). In humans the hematopoietic compartment suffers deregulation of stem cell differentiation programs with aging, resulting in higher myeloid to lymphoid progenitor ratios, an event related to “immunosenescence” [72]. An increase in myeloid/lymphoid ratios is usually indicative of innate immune system activation, suggestive of chronic inflammation [73]. Low levels of RBC and alteration in myeloid/lymphoid ratios surged independently of the presence of short telomeres during WT aging (Fig 4D, S9A and S9C Fig). Of these phenotypes, *tert*^*-/-*^ mutants only anticipated the low levels of RBC (p = 0.0067, Fig 4D and S9A Fig). *tert*^*-/-*^ myeloid/lymphoid and precursors ratios accompanied the tendencies of WT up to 12 months of age (S9A and S9C Fig). We could not detect significant differences in total cell number per blood volume with aging in WT or *tert*^*-/-*^ mutants (S9B Fig). Thus, deregulation of cell differentiation programs in the hematopoietic system that accompanies aging is likely to be independent of kidney marrow telomere-length. Nevertheless, telomere shortening accompanies the decline in RBC levels with age.

### *tert*^*-/-*^ mutants recapitulate diseases of old age

We established that telomere shortening is associated with tissue specific aging phenotypes in zebrafish. Non-tissue specific disease phenotypes also emerged with aging, namely cachexia, infection and cancer. The onset of age-associated pathology correlated with the age at which most tissues started to display short telomeres (fin, gut and muscle, Figs 1B, 2A and 2B). In agreement, we also observed that *tert*^*-/-*^ mutants recapitulated these diseases prematurely.

#### I) Cachexia

**Fig 5:**
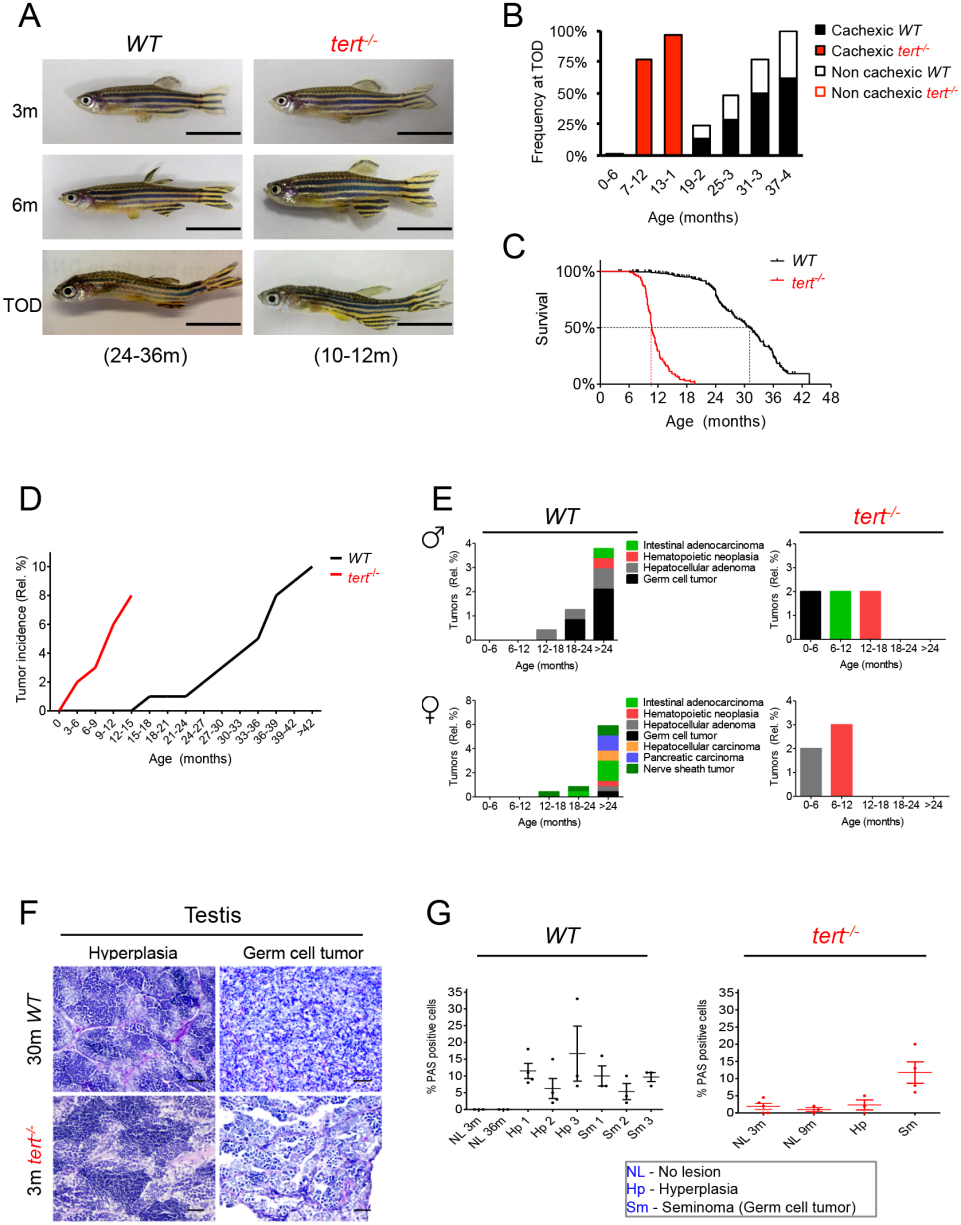
*tert*^*-/-*^ mutants accelerate the onset of age-associated cachexia and neoplasia. A) Representative images of WT and *tert*^*-/-*^ mutants show that, at time-of-death (TOD), zebrafish exhibit signs of cachexia and deformation of the spine, with very low body mass indexes. B) In WT population the incidence of these alterations increases with age, while in *tert*^*-/-*^ mutants, at TOD, 100% of the population is affected. C) Sustained cachexia in older zebrafish is accompanied by an increase in mortality as shown by Kaplan Meier curves of WT and *tert*^*-/-*^ zebrafish. WT zebrafish have a half-life of 30.8m, 3x greater than the half-life of 10.6m of *tert*^*-/-*^ (p<0.0001). N = 426 WT; N = 98 *tert*^*-/-*^, for Fig 5A–5C. TOD corresponds to the interval comprising the second and third quartiles of survival (25 to 75%). Scale bar = 1 cm. D) Tumorigenesis exponentially accelerates with age in both WT and *tert*^*-/-*^ zebrafish, reaching cumulative incidences of ca. 10% and ca. 8%, respectively. N = 23/238 WT; N = 5/66 *tert*^*-/-*^. *tert*^*-/-*^ mutants have an earlier onset of neoplasia starting at 4 months of age (p = 0.003). E) Tumors in WT male zebrafish affect mainly the reproductive system, following the appearance of Periodic Acid Shift (PAS+) stained cells in the tissue stroma (F), (quantified in G). WT females show an even distribution of tumors between reproductive and hematopoietic systems, followed by liver, intestine and pancreas. N = 118 WT males; N = 120 WT females; N = 58 *tert*^*-/-*^ males; N = 8 *tert*^*-/-*^ females. Quantifications of %PAS+ cells was performed in 3 fields of view for each individual in the graph. Scale bar = 50 μm. Data are represented as mean +/- SEM.

Human aging is commonly characterized by weight loss, reduction of subcutaneous fat and sarcopenia, a condition often referred to as cachexia or wasting syndrome. This syndrome has been associated with increased frailty and mortality [74]. In our cohort, we prospectively analyzed 416 WT zebrafish and found the maximum longevity to be of 43.5 months (Fig 5C). From 24 months onwards, we registered an increasing incidence of mortality associated with cachexia and deformation of the spine (from 13% to 62% between 24 and 42 months, Fig 5A and 5B). Cachexia was scored based on specific histopathological changes in the gut, muscle and testis (see description below, S10B Fig). Whereas 38% of older WT zebrafish died without displaying cachexia, the older the animals died, the more likely they were to be cachexic (Fig 5B). All first generation *tert*^*-/-*^ homozygous mutants had significantly shorter lifespan (ca. 3x lower than WT, Fig 5C). This was accompanied by an accelerated incidence of cachexia, from 8 months onwards (Fig 5A and 5B and S10A Fig), a feature that revealed to be common to all *tert*^*-/-*^ zebrafish at time of death. Higher incidence of cachexia and deformation of the spine has been observed by others in older cohorts of zebrafish, by 18–24 months of age [75]. However, the cause of cachexia remains unknown.

Cachexia in both WT and *tert*^*-/-*^ mutants associated with particular tissue alterations in fat, gut, muscle and testis. Aging in humans also associates with defects in lipids storage in subcutaneous adipose tissues and with an increase deposition of visceral fat in skeletal muscle, liver, heart and bone marrow [76]. We observed a loss of subcutaneous and visceral fat mass with WT aging (adipocytes were ~3 times smaller at 36 months vs. 3 months, S11A–S11C Fig), which was significantly more pronounced in cachexic stages (indicated by time-of-death, TOD, in graphs in S11C Fig). In *tert*^*-/-*^ mutants, smaller adipocytes were visible in subcutaneous and visceral tissues by 12 months (S11A–S11C Fig). In cachexic zebrafish, we saw a higher exhaustion of fat reserves when comparing with non-cachexic *tert*^*-/-*^ mutants (S11A–S11C Fig). In the gut, we found marked decrease of villi length (S10B Fig). Accordingly, intestinal crypt depletion and villus atrophy was previously observed in late generation *tert* KO mice [31]. The muscle showed a higher degree of atrophy associated with myocyte degeneration and endomysial edema than that observed in non-cachexic zebrafish during aging (S10B Fig). Testis also showed a more pronounced atrophy with reduced germ cell compartment, when compared with age-matched non-cachexic zebrafish (S10B Fig).

To test if gut villi and muscle degeneration were direct consequences of poor nutritional states in old WT and *tert*^*-/-*^ mutants, 12-month old WT zebrafish were put on a caloric restriction diet for 8 weeks, consisting of transition from *ad libitum* feeding regimen to a food intake reduced by 85%. We observed these zebrafish on caloric restriction displayed width/length ratios similar to cachectic zebrafish (S10A Fig), maintained villi length within the normal range but showed morphological changes in the muscle similar to those described for aging WT fish (S12 Fig). Thus, while aging-related sarcopenia could potentially be triggered by a poor nutritional state the same does not apply to gut villi length. Altogether, our results show that terminal *tert*^*-/-*^ degeneration recapitulates within 12 months the macroscopic phenotypes of old WT zebrafish.

#### II) Swim bladder infection

We observed an increasing incidence of swim bladder infection with age (aerocystitis). By 36 months of age, up to 30% of WT population showed aerocystitis (indicated by black arrow, N = 71 out of a total of 238 zebrafish analyzed, S13A and S13B Fig), the majority of which concomitant with cachexia. 3% of *tert*^*-/-*^ mutants showed swim bladder infection (N = 2 out of a total of 66 zebrafish analyzed, S13A and S11B Figs) and always in association with cachexia. Our analyses suggest that bacterial aerocystitis is a prime candidate for cause of death in aged WT and *tert*^*-/-*^ mutants. Bacterial and fungal infections are known to typically affect the swim bladder via the pneumatic duct in different fish species [77,78]. Thus, concomitant with the appearance of cachexia, *tert*^*-/-*^ mutants showed a tendency to anticipate the rise of infections observed during zebrafish aging.

#### III) Cancer

Cancer is an age-associated disease relying on mechanisms–such as accumulation of DNA damage–that emerge with time [79]. Like other diseases of aging, human cancer incidence rises exponentially after mid-life [80]. Telomere maintenance is a key factor for tumor progression and most commonly achieved via telomerase reactivation [12]. Telomerase expression and activity are upregulated in 90% of human cancers [81]. In order to explore the role of telomerase in tumorigenesis in zebrafish, we analyzed 66 *tert*^*-/-*^ mutants versus 238 WT zebrafish for the incidence of spontaneous tumors. Zebrafish tumors were reported to develop spontaneously at a rate of 11% [75,82] and to resemble human tumors, in their histological and gene expression profiles [83]. Accordingly, we observed a spontaneous tumor cumulative incidence of 10% in WT zebrafish up to 42 months of age (N = 23 in a total of 238 zebrafish analyzed; Fig 5D). Surprisingly, even though *tert*^*-/-*^ displayed approximately the same incidence, 8% (N = 5 in a total of 66 zebrafish analyzed; Fig 5D), they had a much earlier onset, at the age of 4 months (when compared with 24 months in WT; p = 0.003; Fig 5D). 30% of all WT tumors (N = 7/23), including leukemia (N = 1/23) intestinal adenocarcinoma (N = 3/23) hepatocellular carcinoma (N = 1/23) and peripheral nerve sheath tumor (N = 2/23), and 40% of all *tert*^*-/-*^ tumors, specifically leukemia (N = 2/23), were invasive.

Consistent with previous studies [75], male tumors arising with aging in zebrafish comprised mainly neoplasias of the reproductive tissue (Fig 5E) but none of these tumors showed invasive capacities. Tumor development in the gonads generally followed gross enlargement of the organ (with hyperplasia of spermatogonia, S7C Fig) and the appearance of inflammatory Periodic Acid Shift (PAS+) stained cells in the tissue stroma (Fig 5F). Cells with PAS+ staining are associated with the activation of innate and adaptive immune responses in zebrafish [84–87]. We found the appearance of PAS+ cells to be specific to pre-cancerous and cancerous lesions, since healthy testis in different time points exhibited only 0–2% of these cells (Fig 5G). Thus, a boost in inflammatory response appears to precede cancer development in zebrafish gonads. Whether inflammation is promoting cancer or is a protective (albeit insufficient) response mechanism remains to be tested.

Liver and intestinal tumors were also observed in older WT and in *tert*^*-/-*^ male zebrafish (Fig 5E). WT females (N = 120) showed a higher heterogeneity in their tumor spectra, with an even distribution of neoplasia between the reproductive and hematopoietic system, liver, intestine and pancreas (Fig 5E). In order to confirm that tumorigenesis in *tert*^*-/-*^ was not due to a possible reversion of the telomerase point mutation, we genotyped 3 tumors (out of the total of 5 tumors) and confirmed that the mutation background was maintained. Thus, telomerase does not appear to be a limiting factor for initiation of tumorigenesis in zebrafish, consistent with short telomeres triggering more microadenomas in CAST/EiJ mice [25]. Telomerase absence does not significantly change incidence/malignancy rates but accelerates the onset of zebrafish cancer. The appearance of DDR could imply that early genome instability events precede activation of alternative mechanisms for elongation of telomeres.

## Discussion

In humans, telomere erosion has been implicated in age-associated diseases [26,88,89]. However, it is not clear whether telomere shortening is an actual cause of aging and how it contributes towards the development of degenerative phenotypes. The identification of tissues where intrinsic telomere shortening becomes truly limiting for optimal function and homeostasis is crucial for understanding the impact of short telomeres in aging. Moreover, understanding whether tissue specific telomere shortening triggers systemic aging signals will help develop an integrative model of how telomere length influences the aging process as a whole.

In our current work, we show that absence of telomerase accelerates diseases of old age, including cancer. We find that zebrafish telomerase mutants show an earlier cancer onset and maintain a close to 40% tumor invasion rate. This is surprising since shutting off telomerase is thought to have evolved as a tumor suppressor mechanism by limiting cell proliferation. How tumors in *tert*^*-/-*^ zebrafish succeed in stabilizing chromosome-ends in the absence of telomerase is currently unknown. The simplest explanation would entail the engagement of ALT mechanism, usually activated in the absence of telomerase [90,91]. It is possible that the effectiveness of ALT may vary depending on different genetic backgrounds. Absence of telomerase in mouse models may either decrease or increase tumorigenesis depending on the genetic context [33,92–94]. Our data shows that, instead of grossly altering tumor incidence, short telomeres accelerate the appearance of spontaneous tumors to juvenile stages in zebrafish, in agreement with previous studies [95].

Accumulation of short telomere induced damage anticipates dysfunction in specific tissues with natural aging. Consistently with telomere shortening affecting mainly proliferative tissues [29,30,32], the gut appears as a leading candidate-organ where telomeres shorten over time reaching telomerase mutant length, our reference for critically short telomeres. However, similarly to the gut, the muscle also accumulates short telomeres and DDRs with age, which anticipate severe sarcopenia phenotypes. The rate of telomere shortening in human somatic tissues is not exclusively dictated by the respective rates of proliferation [51]. Reactive oxygen species (ROS) cause increased DNA lesions at telomeres that result in telomere shortening [55,58,96,97]. Our data show that ROS levels increase by ca. 12 times with WT zebrafish aging specifically in the muscle, and not in the gut. Thus, whereas cell proliferation probably acts as a primary trigger for telomere shortening in the gut, increased levels of oxidative damage may underlie the decline in muscle telomere length over time. Accordingly, less proliferative tissues of TERT knockout mice also have increased production of ROS due to mitochondrial dysfunction [34]. Alternatively, telomere shortening without significant cell division over time in the muscle could rely on Telomere Rapid Deletion (TRD) mechanisms, as previously observed in mammalian cells. Interestingly, both gut and muscle follow a similar behavior of linear telomere decline until 24 months of age with stabilization of length at later ages. Telomere stabilization in older ages has been reported by others [9,11] and goes in hand with the existence of selection processes that may favor the elimination of cells with extremely short telomeres. Alternatively, maintenance of shorter telomere lengths at older zebrafish ages may rely on recombination-based ALT mechanisms. Telomere length maintenance by recombination, first described for yeast cells [98], has been recently found active in mammalian cells, even in the presence of telomerase [99,100]. As shown by increased levels of telomeric sister-chromatid exchange (T-SCEs) and variation of pq ratios, subtelomeric or telomeric recombination is increased in short telomere cells from telomerase positive mice, Dyskeratosis Congenita patients and in human cancer lines [99,100].

Telomeres in the testis and kidney marrow do not reach the critical length of telomerase mutant zebrafish. It, thus, appear that short telomeres do not anticipate these tissues’ dysfunction with aging. We cannot exclude, however, that a minority of cells in these tissues accumulates short telomeres with age and these cannot be detected by methods of whole population analysis. The lack of significant detectable telomere shortening in the testis is consistent with previous work showing that zebrafish gonads retain high telomerase activity levels [47]. Interestingly, testis is the tissue with the highest incidence of cancer in zebrafish, and telomerase is known to be re-activated in 90% of all human cancers [81]. The significant expansion of immature germ cells (spermatogonia, spermatocytes) in detriment of mature spermatozoa in WT testis with age could also explain why we do not detect telomere shortening in this tissue. Furthermore, this dramatic alteration in cellular composition may lead to changes in free testosterone levels, which associate with loss of muscle mass during human aging [101]. In contrast, the kidney marrow is the only tissue where significant telomere elongation occurs during development and sexual maturation of zebrafish, from larvae to 3 months. From all WT tissues tested in adult stages, the kidney marrow shows the longest telomeres and the biggest difference between WT and *tert*^*-/-*^ telomere length. However, kidney marrow *tert*^*-/-*^ telomeres have lengths within the range of other *tert*^*-/-*^ tissues. Thus, at least during development, the zebrafish kidney marrow probably has a higher necessity for telomerase activity than other tissues and perhaps retains it in some cells, as in the human hematopoietic compartment [102]. Telomeres still shorten in the kidney marrow with age after 18 months, even though they do not reach telomerase mutant lengths. These data suggest that, similarly to humans, telomere shortening in certain tissues and cell types probably relates to differential restriction of telomerase expression [8].

Tissues where telomeres do not reach telomerase mutant lengths still develop aging phenotypes, namely decline in fertility and anemia. It does not seem likely, however, that these phenotypes arise in a completely telomere-independent manner since they are recapitulated prematurely by telomerase mutants. Thus, aging phenotypes in testis and kidney marrow are due either to: 1) non cell-autonomous effects caused by telomere shortening in the gut, muscle and/or unidentified tissues or 2) absence of non-canonical functions of telomerase required for proper tissue homeostasis, or a combination of both effects. Regarding the first point, as telomeres shorten the number of senescent cells increase in tissues. These cells may be secreting SASP molecules, such as IL-6 and IL-8 which have been described as capable of influencing growth in neighboring cells and epithelial–mesenchymal transition non cell autonomously [36]. However, non-catalytic telomerase functions have also been described as important determinants of tissue physiology in mammals and zebrafish [103–105]. Emerging evidence implicates a role for telomerase in stimulation of cell proliferation and regeneration, independently of telomere length maintenance, via activation of Wnt signaling [106]. Particularly in zebrafish embryos, knockdown of *tert*, *terc* or *dkc1* was shown to trigger larval phenotypes that are reminiscent of human DC, such as pancytopenia, impaired hematopoietic differentiation or p53-dependent apoptosis [104,105,107]. Importantly, these larval premature degenerative phenotypes also develop independently of changes in telomere length [104,105,107]. Parallel to this work, the decline in cell proliferation, increased apoptosis and anemia we observe in WT aging and in *tert*^*-/-*^ tissues could be partly attributed to non-canonical functions of telomerase, as demonstrated to occur in zebrafish larvae. Finally, the observed defects in testis and kidney marrow could still rely on loss of telomere integrity with age that is not detectable using the assays we employed. Alternatively, we cannot exclude that aging associated dysfunction in these tissues (testis and kidney marrow) occurs independently of changes in telomere length. In the future it will be important to dissect the mechanisms by which telomere shortening induced stress in one tissue is perceived by other tissues in the rest of the organism.

**Fig 6:**
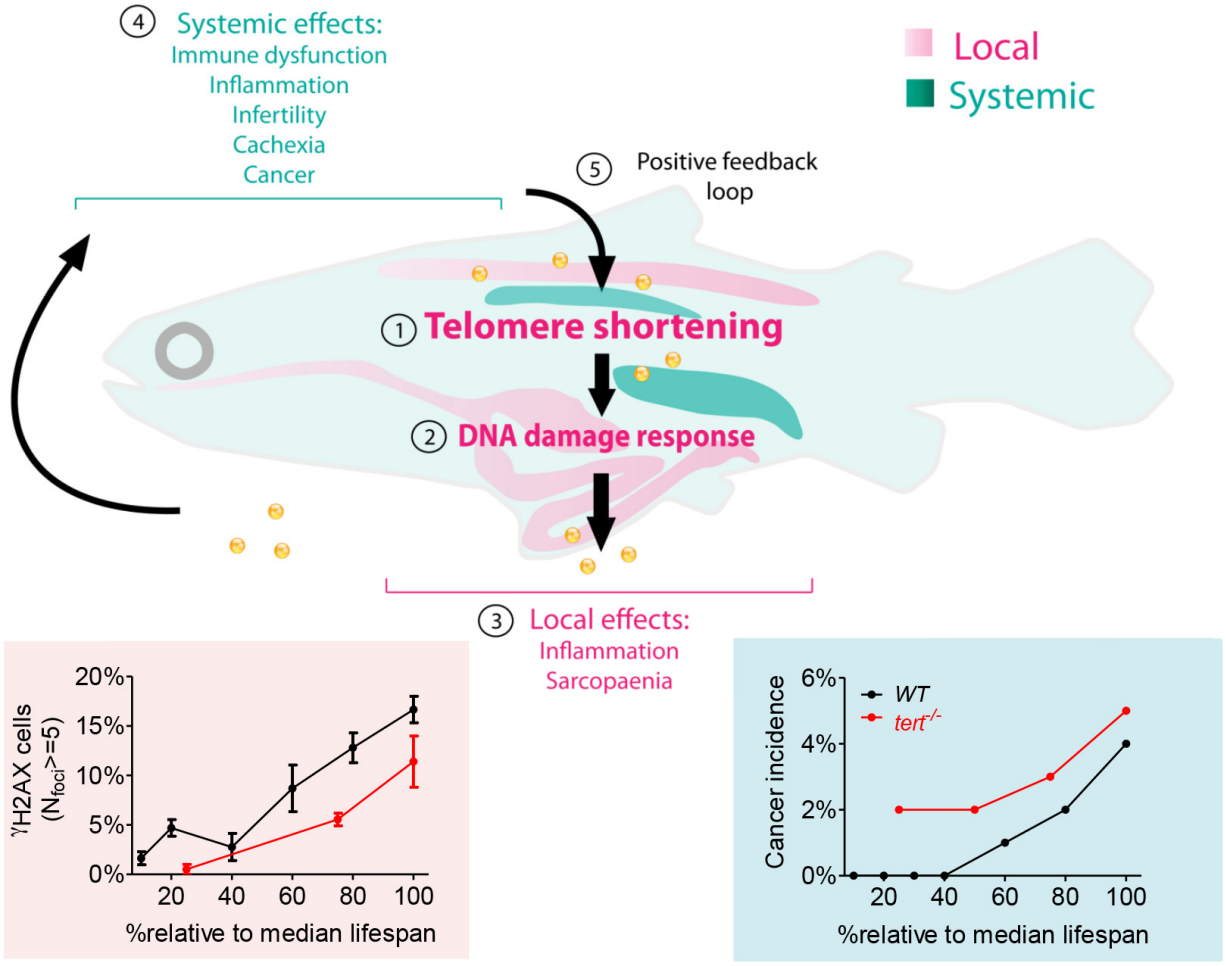
Working model—short mTL and DDR in key tissues establish the rate of local and systemic aging. Expression of telomerase is restricted in most somatic tissues resulting in telomere shortening with aging (1). In gut and muscle, shortening of telomeres to critical levels results in increased DDRs with aging (2) which disrupt local homeostasis, culminating in organ and tissue-specific lesions such as inflammation and sarcopenia (3). Gut and muscle DDRs act in a cell non-autonomous manner by inducing defects in organs where telomeres do not shorten appreciably, such as testis and kidney marrow (4). DDR signals trigger systemic damage resulting in cachexia and fueling tumorigenesis. Overall, cellular damage creates an amplifying positive feedback loop accelerating tissue dysfunction with aging (5).

Our work shows that telomere shortening and accumulation of DDR in particular tissues anticipates aging associated dysfunction. As previously proposed [108], accumulation of short telomeres and high levels of persistent DDR in one specific tissue (gut) may be enough to establish the rate of aging in other tissues by non cell autonomous mechanisms (Fig 6). Strikingly, absence of telomerase triggers premature recapitulation of aging phenotypes in most tissues, even in those where telomeres do not reach *tert*^*-/-*^ shortened telomere lengths during aging. Importantly, by using the “percentage of life” instead of absolute age to measure the progression of aging phenotypes, we find surprisingly similar kinetics between the dynamics of WT and *tert*^*-/-*^ phenotypes over time, local and systemic (Fig 6). Thus, rescuing telomere length of aged zebrafish in key tissues (such as the gut) may prove sufficient to restore local and systemic homeostasis in old animals. Whether systemic aging signals will be dominant over locally rejuvenated tissues remains to be tested. However, such therapies will likely only be productive before telomeres reach critical length. Previous studies have shown that reactivation of telomerase in tissues that have undergone extensive telomere erosion is a strong potentiator of tumorigenesis and malignancy [109]. Our study suggests a potential therapeutic window of opportunity, in a period that correlates with mid-life in zebrafish, before age-associated defects become apparent. Time- and tissue-dependent telomerase reactivation will enable us to define and test such a useful period for telomerase therapies.

## Materials and Methods

### Ethics statement

All Zebrafish work was conducted according to National Guidelines and approved by the Ethical Committee of the Instituto Gulbenkian de Ciência and the DGAV (Direcção Geral de Alimentação e Veterinária, Portuguese Veterinary Authority).

### Zebrafish lines and maintenance

Zebrafish were maintained in accordance with Institutional and National animal care protocols. The telomerase mutant line *tert*^*AB/hu3430*^, generated by N-Ethyl-N-nitrosourea (ENU) mutagenesis (Utrecht University, Netherlands; Wienholds, 2004), has a T-A point-mutation in the *tert* gene. *tert*^*AB/hu3430*^ line (available at the ZFIN repository, ZFIN ID: ZDB-GENO-100412-50, from the Zebrafish International Re-source Center—ZIRC). The protocols used for outcrossing mutagenized male zebrafish were previously described [30]. The *tert*^*hu3430/hu3430*^ homozygous mutant (*tert*^*-/-*^) was obtained by incrossing our *tert*^*AB/hu3430*^ strain. WT siblings were used as controls. Genotyping was performed by PCR of the *tert* gene followed by sequencing [30]. Overall characterization of *tert*^*-/-*^ and WT zebrafish was performed in F1 animals produced by *tert*^*+/-*^ incross. Due to a male sex bias in our crosses that affected mostly *tert*^*-/-*^ progeny, we were unable to obtain significant numbers of females for analysis and so the majority of our data is restricted to males unless otherwise stated.

All animals showing signs of morbidity that persisted for up to 5 days, such as inability to eat or swim, or macroscopic lesions/tumors were sacrificed in 200 mg/L of MS-222 (Sigma, MO, USA).

### Telomere restriction fragment (TRF) analysis by Southern blot

TRF analysis was performed as previously described [54]. Briefly, genomic DNA was extracted from freshly isolated tissue using lysis buffer (Fermentas K0512) supplemented with 1 mg/ml Proteinase K (Sigma, MO, USA) and RNase A (1:100 dilution, Sigma, MO, USA). Samples were incubated at 50°C for 18 h in a thermomixer and genomic DNA was extracted by equilibrated phenol-chloroform (Sigma, MO, USA) and chloroform-isoamyl alcohol extraction (Sigma, MO, USA). Genomic DNA was quantified and normalized so the same amount of DNA was digested with RSAI and HINFI enzymes (NEB, MA, USA) for 12 h at 37°C. Samples were electrophoresed on a 20 cm 0.6% agarose gel, in 0.5% TBE buffer, at 4°C for 17 h at 110 constant voltage. A 1.6 kb telomere probe, (TTAGGG)n, labelled with [α-32P]-dCTP using the Prime-it II random primer labelling kit (Stratagene) was used for Southern blotting. 3–6 different WT and *tert*^*-/-*^ individuals belonging to different age groups were used for quantification of each Southern Blot experiment.

### Detection of intracellular oxidant activity

Reactive oxygen species (ROS) accumulation was assessed by measuring the levels of the oxidized form of the cell-permeant 5-chloromethyl-2’,7’- dichlorodihydrofluorescein diacetate (DCFDA) (Sigma). Briefly, zebrafish were euthanized with MS-222 and gut and muscle were dissected. Each tissue was homogenized in 100 μl of ROS buffer (0.32 mM sucrose, 20mM hepes, 1mM MgCl2 and 0.5mM Phenylmethanesulphonyl fluoride). Homogenates were centrifuged and 20 μl of the supernatant was transferred to a 96-well plate and incubated in 1 μg/ml of DCFDA for 30 minutes. Fluorescence values were measured with a Victor 3 plate reader and normalized to total protein content. N = 3 per time point.

### Histology and phenotypic analysis

Zebrafish were sacrificed by anesthetic overdose, in 200 mg/L of MS-222 (Sigma, MO, USA), fixed for 72 hours in 10% neutral buffered formalin and decalcified in 0.5 M EDTA for 48 h. Whole fish were then paraffin-embedded and 3 micrometer midline sagittal sections were stained with hematoxylin and eosin for histopathological analysis. Sections were examined by a pathologist (TC), blinded to experimental groups and microphotographs were acquired in a Leica DM2500 microscope coupled to a Leica MC170 HD microscope camera. A total of 238 WT and 66 *tert*^*-/-*^ zebrafish were submitted for histopathological analysis. At least 4 animals from each age group/genotype were analyzed.

### Immunofluorescence (IF) and confocal analysis

Sections were deparaffinized, rehydrated and heat-induced antigen retrieval was performed in citrate buffer (10 mM Sodium Citrate, pH 6) for 20 minutes at 103°C in an oven. Slides were left to cool down for 30 minutes at room temperature (RT), washed three times in dH20 for 5 minutes each and blocked for 1 hour at RT in 0.25% BSA in PBST (Triton 0.3%). The following primary antibodies were used: rabbit polyclonal antibodies against Proliferation Cell Nuclear Antigen (PCNA, Santa Cruz, CA, USA, 1:50 dilution) and Histone H2A.XS139ph (GTX127342, USA, 1:500). Incubation with primary antibodies was performed overnight at 4°C, followed by three 10 minute PBS washes. Incubation with the secondary antibody Alexa Fluor 568 goat anti-rabbit (Invitrogen, UK, 1:500 dilution) overnight at 4°C was followed by three 10 minute PBS washes, DAPI staining (Sigma, MO, USA) and mounting with DAKO Fluorescence Mounting Medium (Sigma, MO, USA). Apoptosis was detected using the *In Situ* Cell Death Detection Kit (Roche, SW) according to manufacturer's instructions. Briefly, deparaffinized slides were incubated with 40 μg/ml Proteinase K in 10 mM Tris-HCl pH 7.4, 45 minutes at 37°C. Slides were washed in 2×5 minutes in PBS and then incubated with TUNEL labelling mix (protocol indicated by the supplier). Washing and mounting were performed as previously described. Images were acquired on Leica SP5 Live Upright confocal microscope (Leica Microsystems, GER) equipped with Leica Las AF Lite software and with appropriate configurations for multiple color acquisition. For quantitative and comparative imaging, equivalent image acquisition parameters were used. The percentage of positive nuclei was determined by counting a total of 500–1000 cells per slide, 63x amplification (N = 3–4 zebrafish per time point/genotype).

### γH2aX immunofluorescence-Telomere FISH (Fluorescence In Situ Hybridization)

Zebrafish were sacrificed by anesthetic overdose, in 200 mg/L of MS-222 (Sigma, MO, USA). Gut and testis were dissected and placed in 100 μL of DMEM TrypLE supplemented with 10%FBS. Tissues were digested at 37°C for 1 hour and fixed in an equal volume of 10% buffered formalin with 0.2% Triton for 2 hours at room temperature, agitating. Tissue cells were cytospinned for 5 minutes at 1000 rpm and slides were left drying overnight at room temperature. Slides were washed in 1xPBS/0.1%Triton for 4x5min and incubated in 3%H_2_O_2_ for 20min at 37°C. After washing 3 times for 5 minutes in H_2_O, slides were incubated in acetone at -20°C for 7 minutes. Slides were washed 2 times for 5 minutes and blocked 1 hour at room temperature with PBDX buffer with goat serum (50 mL 1xPBS, 0.5g BSA, 0.5 mL DMSO, 250 μl Triton 10%, 750 μl goat serum). Primary antibody (Histone H2A.XS139ph, GTX127342, USA, 1:100) was added for 1 hour at room temperature and left incubating overnight at 4°C. Primary antibody was washed with 1x PBS/0.1% Triton two times, 10 minutes each, followed by four washed (of 30 minutes each) with PBDX. Secondary antibody Alexa Fluor 568 goat anti-rabbit (Invitrogen, UK, 1:500 dilution) together with DAPI (Sigma, MO, USA, 1:1000) were added to slides for 1 hour at room temperature followed by incubation overnight at 4°C. Secondary antibody was washed with 1xPBS/0.05% Tween four times, 15 minutes each wash, followed by fixation in buffered formalin 10% for 20 minutes. Slides were washed in 1xPBS/0.05% Tween 2x5 minutes and air-dried. Denaturing was performed for 15 min at 80°C in hybridization buffer (70% formamide (Sigma), 25 mM MgCl2, 1 M Tris pH 7.2, 5% blocking reagent (Roche)), containing 3 μg/ml Cy-3-labelled telomere specific (CCCTAA) peptide nuclei acid probe (Panagene), followed by hybridization for 2 h at room temperature in the dark. The slides were washed with 70% formamide in 2×SSC for 10 minutes, followed by two 10 minutes wash with 2×SSC and PBS. Slides were mounted as previously described and imaged in a Leica HCScreening (100x objective). For quantitative and comparative imaging, equivalent image acquisition parameters were used. The percentage of positive nuclei was determined by counting a total of 50–100 cells per slide (N = 3 zebrafish per time point/genotype).

### Senescence-associated β-galactosidase assay

β-galactosidase assay was performed as previously described [110]. Briefly, sacrificed zebrafish adults were fixed for 72h in 4% paraformaldehyde in PBS at 4°C and then washed three times for 1 h in PBS-pH 7.4 and for a further 1 h in PBS-pH 6.0 at 4°C. β-galactosidase staining was performed for 24 h at 37°C in 5 mM potassium ferrocyanide, 5 mM potassium ferricyanide, 2 mM MgCl2 and 1 mg/ml X-gal, in PBS adjusted to pH 6.0. After staining, fish were washed three times for 5 minutes in PBS pH 7 and processed for de-calcification and paraffin embedding as before. Sections were stained with nuclear fast red for nuclear detection and images were acquired in a bright field microscope (Olympus BH2, LB). To quantify SA-β-GAL staining we used an arbitrary scale of signal intensity (where weaker signal is represented by 1 and stronger signal by 4; N≥3 per time point per genotype).

### Fertility assays

Fish were kept in their living tanks until they were randomly selected for breeding. Briefly, breeding pairs were left overnight in external breeding tanks and collection of eggs was performed the following morning. 3–10 independent breeding trials were conducted for each age group and genotype (using either 6-month old female or male controls, tested previously for successful reproductive ability), to evaluate how aging affects male or female reproduction. Only successful breeding trials, defined as events where a female lays a clutch of eggs when paired with a male, were scored. In every trial group, a breeding box including a 6-month old couple was included to ensure the protocol in place guarantees maximum reproductive output.

Embryos were collected approximately 2 hours post fertilization (hpf) and allowed to develop at 28°C. Assessment of egg fertilization and embryo viability was conducted between 2 and 4 hpf.

### Flow cytometry and cytology of head kidney marrow

Single-cell suspensions from head kidney marrow (HKM) were prepared from adult zebrafish. Freshly isolated head kidney tissue was placed in a 1.5ml eppendorff containing 200μl of complete high-glucose DMEM (10% FBS; 1% pen/strep) on ice. Tissue was maintained in the tube, on ice, while gently disrupted manually with the aid of a plastic pestle. Single-cell suspensions were obtained by gentle pipetting and filtering through a 40μM nylon membrane cell strainer (Falcon). Cells in complete DMEM were incubated for 15’ on ice with 1mg/ml propidium iodide for dead cell exclusion analysis.

Single cell suspensions from HKM were examined using a CyanADP flow cytometer (Beckman Coulter), using PI for dead cell exclusion and SSC VS FSC to identify the different cell populations, as previously described [85]. Data were analyzed using FlowJo software Version 9.4.11 (TreeStar). N = 3–5 zebrafish per time point per genotype.

### Statistical and image analysis

Image edition was performed using *FIJI* [111]. Statistical analysis was performed in *GraphPad Prism5*, using two-way ANOVA test with Bonferroni post-correction for all experiments comparing WT and *tert*^*-/-*^ over time. Correlations between phenotypes and telomere size were tested using one-tailed Pearson’s test. Comparison of WT and *tert*^*-/-*^ survival curves was performed using a log-rank (Mantel-Cox) test. Comparison of WT and *tert*^*-/-*^ cancer incidence curves was performed using a Kolmogorov–Smirnov test. Significance of fertility decline in *tert*^*-/-*^ mutants was assessed by a Mann–Whitney’s test. A critical value for significance of p<0.05 was used throughout the study.

## Supporting Information

S1 Fig Kidney marrow telomeres elongate dramatically from 3 weeks to 3 months. A) Representative TRFs of kidney marrow genomic DNA and B) respective densitometries for three 3 week and 3 month-old WT samples (N = 3 per time point). C) mTL quantification showing that average mTL significantly elongates from 3 week old larvae to 3 month old WT kidney marrows. Data are represented as mean +/- SEM.
(TIF)

S2 Fig Telomeres shorten with age in the gut and muscle but not testis of WT zebrafish. A-C) Representative densitometries of restriction fragment analysis of genomic DNA by Southern Blot (relative to TRFs in Fig 2A, 2B and 2C, respectively) for one zebrafish of each age (WT at 3, 12, 24 and 36 months and *tert*^*-/-*^ mutants at 3 and 12 months). Telomere length distributions show telomere length decreases with time in the gut (A) and muscle (B) but not testis (C)—shown for single individuals analyzed in each time point. *a*.*u*. represents arbitrary telomere length units.
(TIF)

S3 Fig ROS levels increase with aging in WT muscle. Representative quantification of ROS levels by 5-chloromethyl-2’,7’-dichlorodihydrofluorescein diacetate (DCFDA) staining 6 months and 36 months WT gut and muscle. ROS levels increase significantly with aging in WT muscle. N = 3 per tissue per time point.
(TIF)

S4 Fig Zebrafish aging is accompanied by an increase in DDR and apoptosis, and by a decrease in proliferation. Quantifications of immunofluorescence signal for DNA damage (YH2AX), proliferation (PCNA) and apoptosis (TUNEL) for testis, gut, kidney marrow and muscle of WT (at 3, 6, 9, 18, 24, 30 and 36 months) and *tert*^*-/-*^ mutant siblings (at 3, 6, 9 and 12 months). The correlation between changes in these molecular markers and telomere length is depicted in Fig 3E.
(TIF)

S5 Fig The number of telomere induced foci (TIF) increases with age in the gut. A) Representative immunofluorescence staining of γ-H2AX foci (green) and telomeres by FISH (red) in cells isolated (cytospin) from the gut of 3, 12 and 36 month-old WT and 3-months *tert*^*-/-*^. Yellow arrowheads point to co-localization of γ-H2AX and telomere signal–TIFs. Compared to 3-months, the number of TIFs increases significantly with age in the gut of 36 months (p<0.0001) and 3 month-old *tert*^*-/-*^ zebrafish (p<0.0001), as shown by quantifications in B). Scale bar = 1 μm. Data are represented as mean +/- SEM. N = 3 individuals, per age per time point per genotype (N = 250 cells).
(TIF)

S6 Fig Increase in senescence with age is anticipated by shorter mTL. A) Representative haematoxylin-eosin and senescence-associated β-galactosidase (SA-β-gal) staining in gut, kidney marrow and testis sections of WT (at 3, 12 and 24 months) and *tert*^*-/-*^ mutant siblings (at 3 and 12 months). A significant increase in SA-β-gal positive cells is seen by 12 months of age in *tert*^*-/-*^ tissues and by 24 months in WT tissues (when comparing both with 3 month-old controls). No staining is observed in the muscle. B) Increase in SA-β-gal staining does not significantly correlate with mTL decline in any of the tissues tested. Grey shaded area identifies the telomeric length at which significant SA-β-gal staining is observed in *tert*^*-/-*^ mutants’ tissues. WT and *tert*^*-/-*^ age groups are indicated in each graph by black and red colored numbers, respectively. Quantifications of SA-β-GAL signal were performed using an arbitrary scale of signal intensity (where weaker signal is represented by 1 and stronger signal by 4). N = 3–6 per genotype per time point. Scale bar = 50 μm. N = 3–6 for tissue mTL telomere length quantifications per genotype per time point (x-axis in graphs of S6B Fig). Data are represented as mean +/- SEM.
(TIF)

S7 Fig Aging-associated enteritis and hyperplasia of spermatogonia develop prematurely in *tert*^*-/-*^ tissues. Quantification of the incidence of intestinal inflammatory cell infiltration (enteritis), gut villi length and hyperplasia of the testis in both WT and *tert*^*-/-*^ mutants with aging. A) As WT and *tert*^*-/-*^ mutants age, there is progressive inflammatory cell infiltration of the *lamina propria* in the gut (particularly after the age of 24 months in WT and from 6 months onwards in *tert*^*-/-*^ mutants). WT show 13% of enteritis incidence by 36 months (N = 30/238) and *tert*^*-/-*^ have 18% incidence by 12 months (N = 12/ 66). B) No differences in gut villi length are found during WT or *tert*^*-/-*^ aging (quantification of 5 different fields of view for 4–5 different individuals per time point per genotype). WT and *tert*^*-/-*^ age groups are indicated in each graph by black and red colored numbers, respectively. C) The percentage of zebrafish with hyperplasia of seminiferous epithelium raises to 3% in 36 months WT cohorts (N = 7/238), accompanied by a progressive decrease in fertilization capacity with age (Fig 4C), but maintains at very low levels in *tert*^*-/-*^ mutants (N = 1/66).
(TIF)

S8 Fig Shorter male mTL and aging inhibit female spawning while female reproductive function is mildly affected with age. A) Fertility in WT male zebrafish declines by ca. 50% at 24 months (Fig 4C), and is accompanied by a reduced ability to stimulate female spawning (average of 61 vs. 166 eggs layed in crosses with 24 month-old vs. 3 month-old males, 3–10 fertilization trials per time point). t*ert*^*-/-*^ mutant males show defects in egg spawning stimulation already by 6 months (average of 62 vs. 166 eggs layed in crosses with 3 month-old *tert*^*-/-*^ vs. 3 month-old WT males, 3–10 fertilization trials per time point). B) Conversely, mild reproductive function defects are seen in 24-month vs. 6-month old WT females (ca. 84% vs. 99% reproductive function, respectively, 3–10 fertilization trials per time point). *tert*^*-/-*^ females show a slight defect (ca. 76%) in the percentage of fertilized eggs by 12 months of age when compared with 6 months (3 fertilization trials per time point). C) Female spawning is not significantly affected by age in WT (3–10 fertilization trials per time point) or *tert*^*-/-*^ female populations (3 fertilization trials per time point).
(TIF)

S9 Fig *tert*^*-/-*^ mutants' myeloid to lymphoid ratios accompany the progression in time found for WT aging. A) Identification of zebrafish kidney marrow cells by flow cytometry and B) quantification of the percentage of lymphocytes, myeloid cells, myeloid/lymphoid ratios and precursors for WT (3, 6, 9, 12, 24 and 33 months) and *tert*^*-/-*^ mutants (3, 6, 9 and 12 months). B) The percentage of WT lymphocytes (“L” gate in A) declines from 30% at 3 months to 10% at 33 months (p = 0.0011). *tert*^*-/-*^ mutants also show a decline from 3 to 12 months (p = 0.0099) which accompanies that of WT zebrafish for the same age interval. The percentage of WT myeloid cells (“My” gate in A) increases gradually from 2% at 3 months to 9% at 33 months (p<0.0001). *tert*^*-/-*^ mutants’ percentage of myeloid cells follows the tendency of WT with age, gradually increasing from 3 to 12 months. Consequently, myeloid/lymphoid ratios increase with age in WT kidney marrow. No significant changes are found in WT and *tert*^*-/-*^ populations of precursors (“P” gate in A) with age. N = 3–5 per time point per genotype.
(TIF)

S10 Fig Cachexia at time of death is associated with lower body mass, gut villi shortening and marked muscle and testis atrophy. A) Quantification of width/length ratios shows that at time of death (TOD), both WT and *tert*^*-/-*^ zebrafish have significantly lower body mass indexes when compared with non-cachexic siblings (N = 4–17 per time point for WT zebrafish and N = 3–7 for *tert*^*-/-*^ mutants). B) Representative hematoxilin and eosin-stained sections of gut, muscle and testis from WT and *tert*^*-/-*^ siblings at TOD. B) Cachexia is associated with shorter villi and/or villous atrophy (defined as flattening of the gut mucosal surface, N = 3–6 per genotype per time point) and severe myocyte atrophy and degeneration (N = 3–6 per genotype per time point), to a higher degree than that found for non-cachexic siblings. Testis also shows pronounced atrophy, with reduced germ cell compartment associated with cachexia (N = 3–4 per genotype per time point). TOD corresponds to the interval comprising the second and third quartiles of survival (25 to 75%). Quantifications were performed in at least 3 different fields of view for each individual. Scale bar = 50 μm. Data are represented as mean +/- SEM.
(TIF)

S11 Fig Subcutaneous adipose tissue progressively disappears with aging whereas visceral reserves maintain until later ages. Representative hematoxilin and eosin-stained sections of subcutaneous (A) and visceral (B) adipose tissue depots of WT (3, 12 and 36 months and TOD) and *tert*^*-/-*^ siblings (3 and 12 months and TOD), and C) quantification of the adipocyte vacuole diameter at different ages. A) WT zebrafish show a progressive loss of the subcutaneous depot, with age, C) accompanied by a reduction in the adipocytes’ vacuole diameter (adipocytes are ~3.3 times smaller at 36 months vs. 3 months). *tert*^*-/-*^ anticipate this phenotype by 12 months of age. With cachexia, both WT and *tert*^*-/-*^ zebrafish show complete exhaustion of the subcutaneous adipose tissue depot (at TOD). B) Visceral adipose tissue (peri-pancreatic) is reduced or absent at 36 months in WT zebrafish (when adipocytes are ~3 times smaller than at 3 months) and by 12 months in WT mutants; and similarly to subcutaneous depot, exhaustion of visceral adipose reserves associates with cachexia. N = 3–7 zebrafish per genotype per time point. Time of death (TOD) corresponds to the interval comprising the second and third quartiles of survival (25 to 75%) Scale bar = 50 μm. Data are represented as mean +/- SEM.
(TIF)

S12 Fig Muscle atrophy, but not gut villi degeneration, is the consequence of a poor nutritional body state in cachexic zebrafish. Representative picture and hematoxilin and eosin-stained sections of 12-month old WT after 8 weeks of caloric restriction, where food intake was reduced by 85%. Upon caloric restriction, gut villi length is maintained within a normal range (when compared with non cachexic 12-month old WT–see Fig 4A) but myocytes show atrophy to a similar degree as seen in cachexic WT and *tert*^*-/-*^ zebrafish. N = 4. Scale bar = 50 μm.
(TIF)

S13 Fig Infection of the swim bladder rises with age and is anticipated in *tert*^*-/-*^ mutants. A) Representative hematoxilin and eosin-stained sections of swim bladder from WT (3, 12, 36 months and TOD) and *tert*^*-/-*^ mutants (3, 12 months and TOD). There is massive destruction and inflammation of the swim bladder at time of death (indicated by black arrow), associated with cachexia, which is accompanied by necrosis and often extended throughout the visceral cavity, both in WT and *tert*^*-/-*^ mutants, compatible with aerocystitis. B) Incidence rates reach 30% in 24 month WT zebrafish (N = 71/238) and 3% in 9-month old *tert*^*-/-*^ mutants (N = 2/66). TOD corresponds to the interval comprising the second and third quartiles of survival (25 to 75%). Scale bar = 50 μm. Data are represented as mean +/- SEM.
(TIF)
