# Supplementary figures and images for: Short Telomeres in Key Tissues Initiate Local and Systemic Aging in Zebrafish
Source: PLoS Genet. 2016 Jan 20;12(1):e1005798. doi: 10.1371/journal.pgen.1005798 (PMC4720274; doi:10.1371/journal.pgen.1005798)

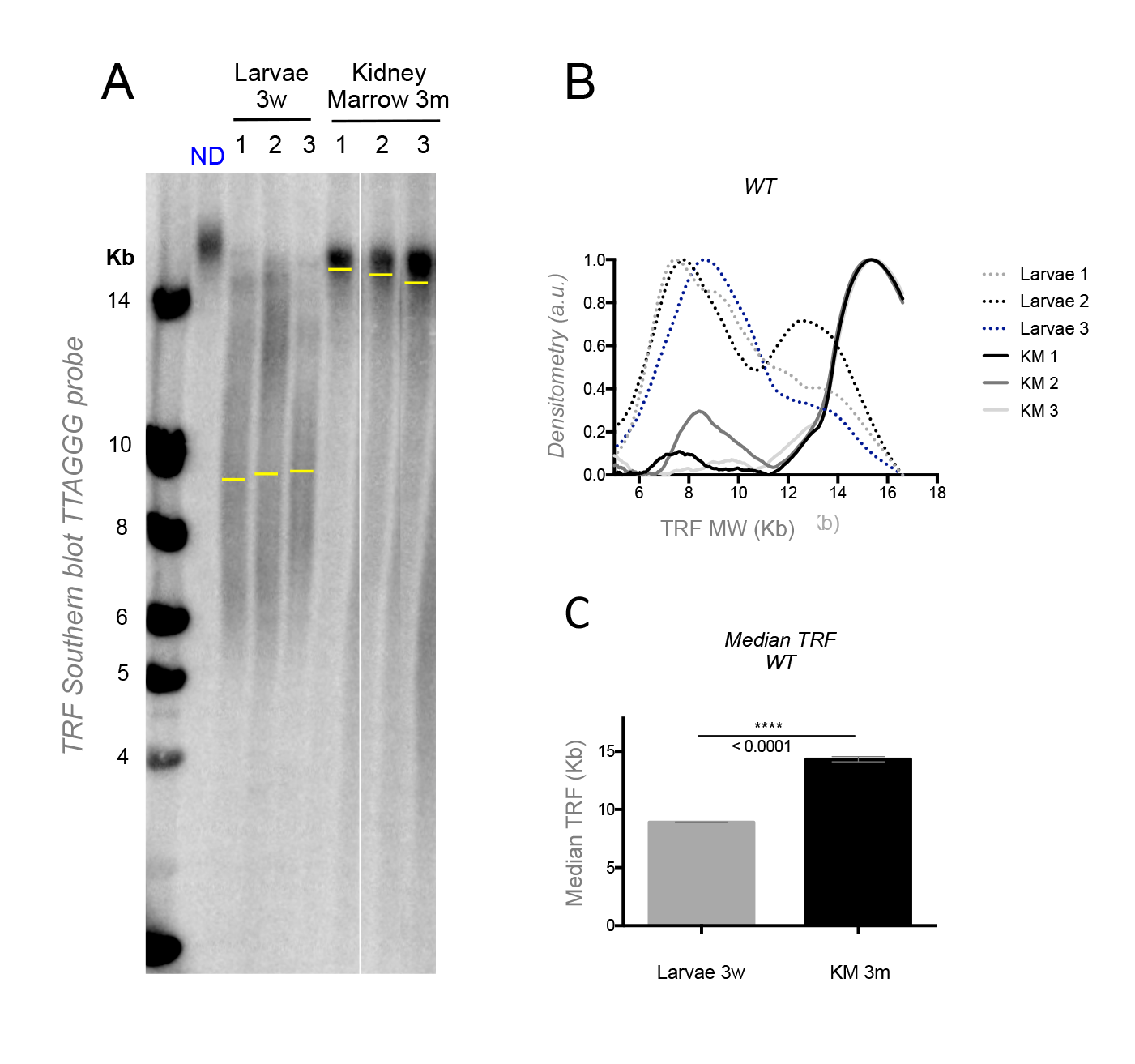

Supplement: S1 Fig — A) Representative TRFs of kidney marrow genomic DNA and B) respective densitometries for three 3 week and 3 month-old WT samples (N = 3 per time point). C) mTL quantification showing that average mTL significantly elongates from 3 week old larvae to 3 month old WT kidney marrows. Data are represented as mean +/- SEM. (TIF) [file pgen.1005798.s001.tif]

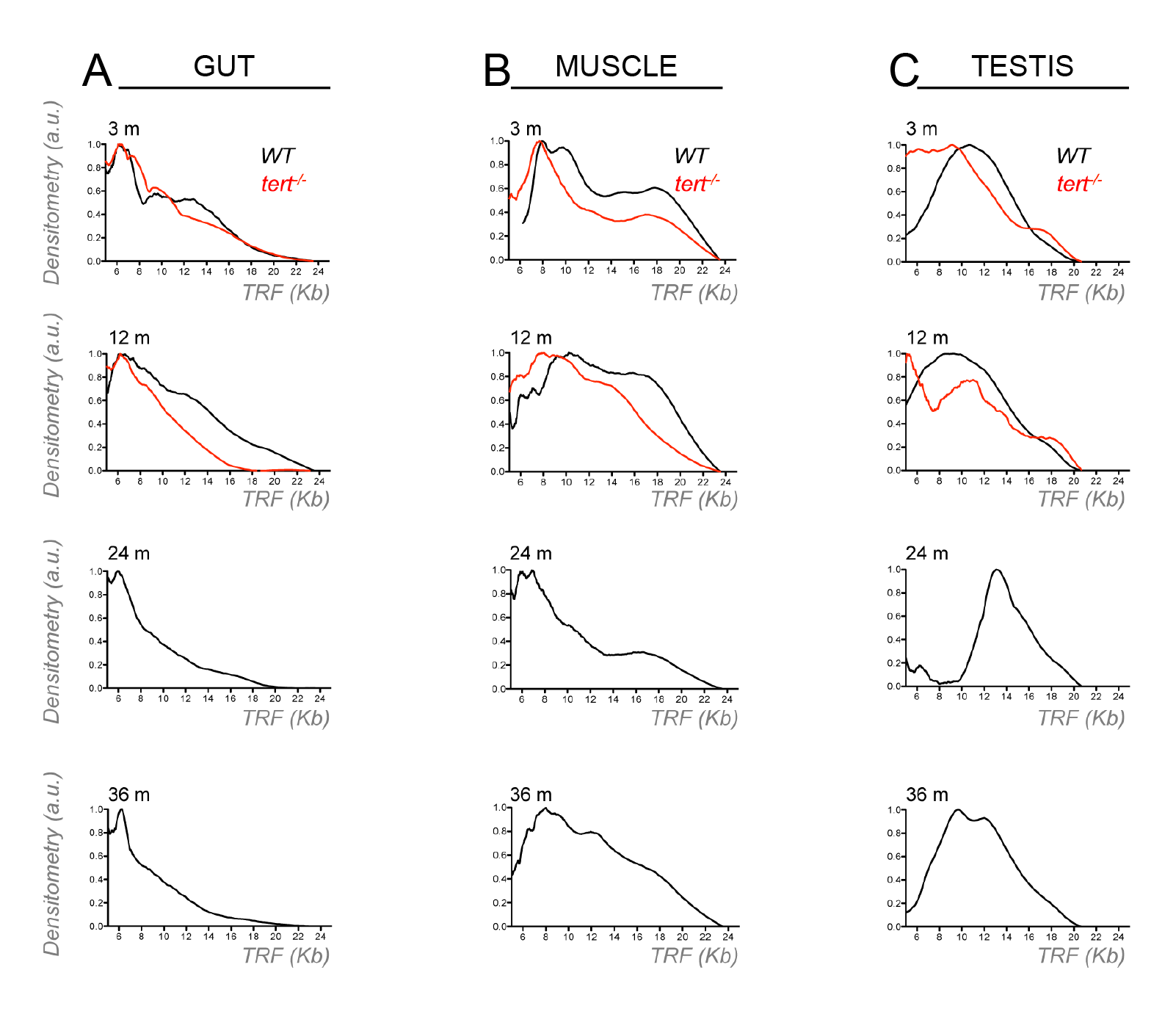

Supplement: S2 Fig — A-C) Representative densitometries of restriction fragment analysis of genomic DNA by Southern Blot (relative to TRFs in Fig 2A, 2B and 2C, respectively) for one zebrafish of each age (WT at 3, 12, 24 and 36 months and tert-/- mutants at 3 and 12 months). Telomere length distributions show telomere length decreases with time in the gut (A) and muscle (B) but not testis (C)—shown for single individuals analyzed in each time point. a.u. represents arbitrary telomere length units. (TIF) [file pgen.1005798.s002.tif]

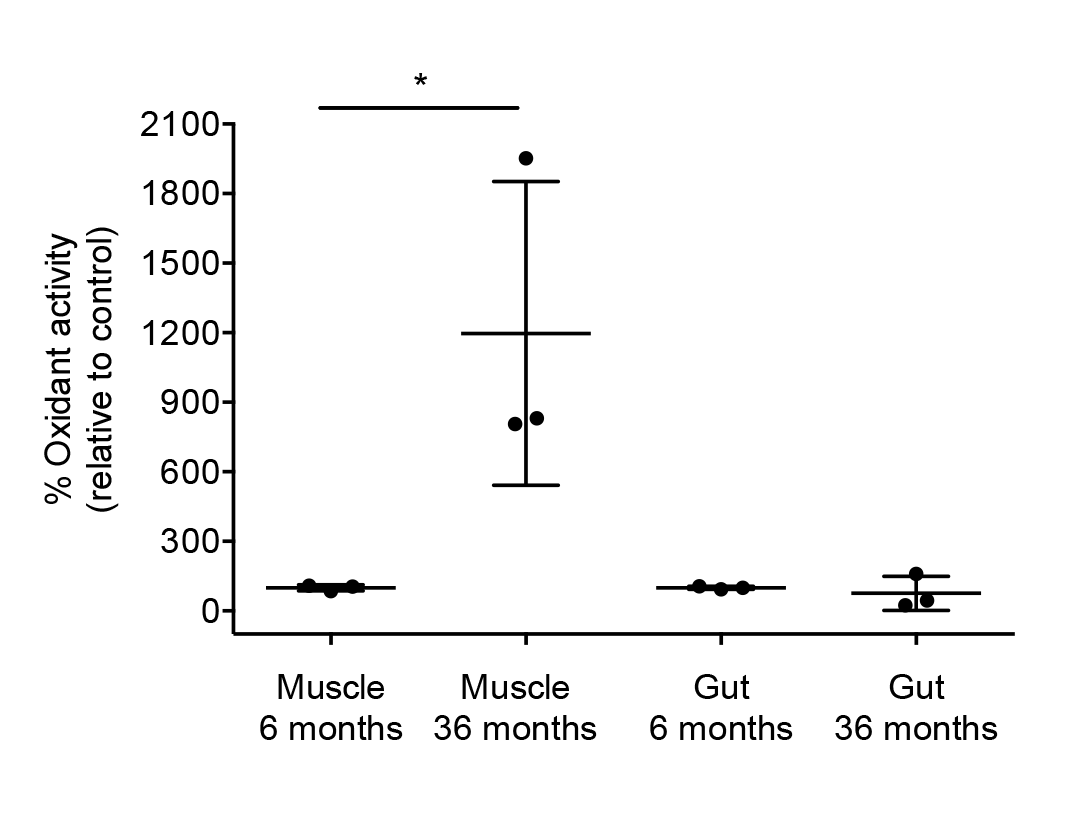

Supplement: S3 Fig — Representative quantification of ROS levels by 5-chloromethyl-2’,7’-dichlorodihydrofluorescein diacetate (DCFDA) staining 6 months and 36 months WT gut and muscle. ROS levels increase significantly with aging in WT muscle. N = 3 per tissue per time point. (TIF) [file pgen.1005798.s003.tif]

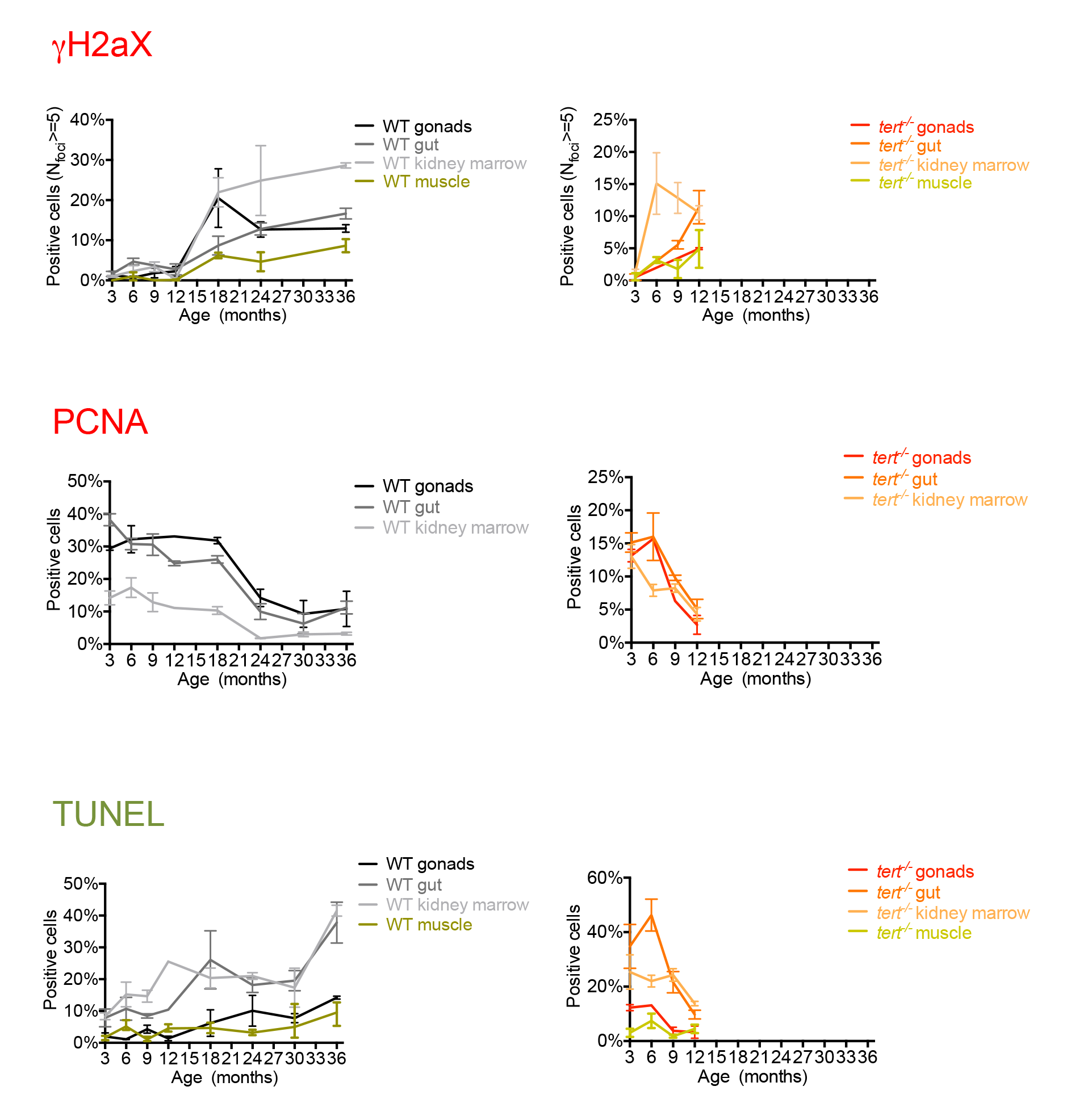

Supplement: S4 Fig — Quantifications of immunofluorescence signal for DNA damage (YH2AX), proliferation (PCNA) and apoptosis (TUNEL) for testis, gut, kidney marrow and muscle of WT (at 3, 6, 9, 18, 24, 30 and 36 months) and tert-/- mutant siblings (at 3, 6, 9 and 12 months). The correlation between changes in these molecular markers and telomere length is depicted in Fig 3E. (TIF) [file pgen.1005798.s004.tif]

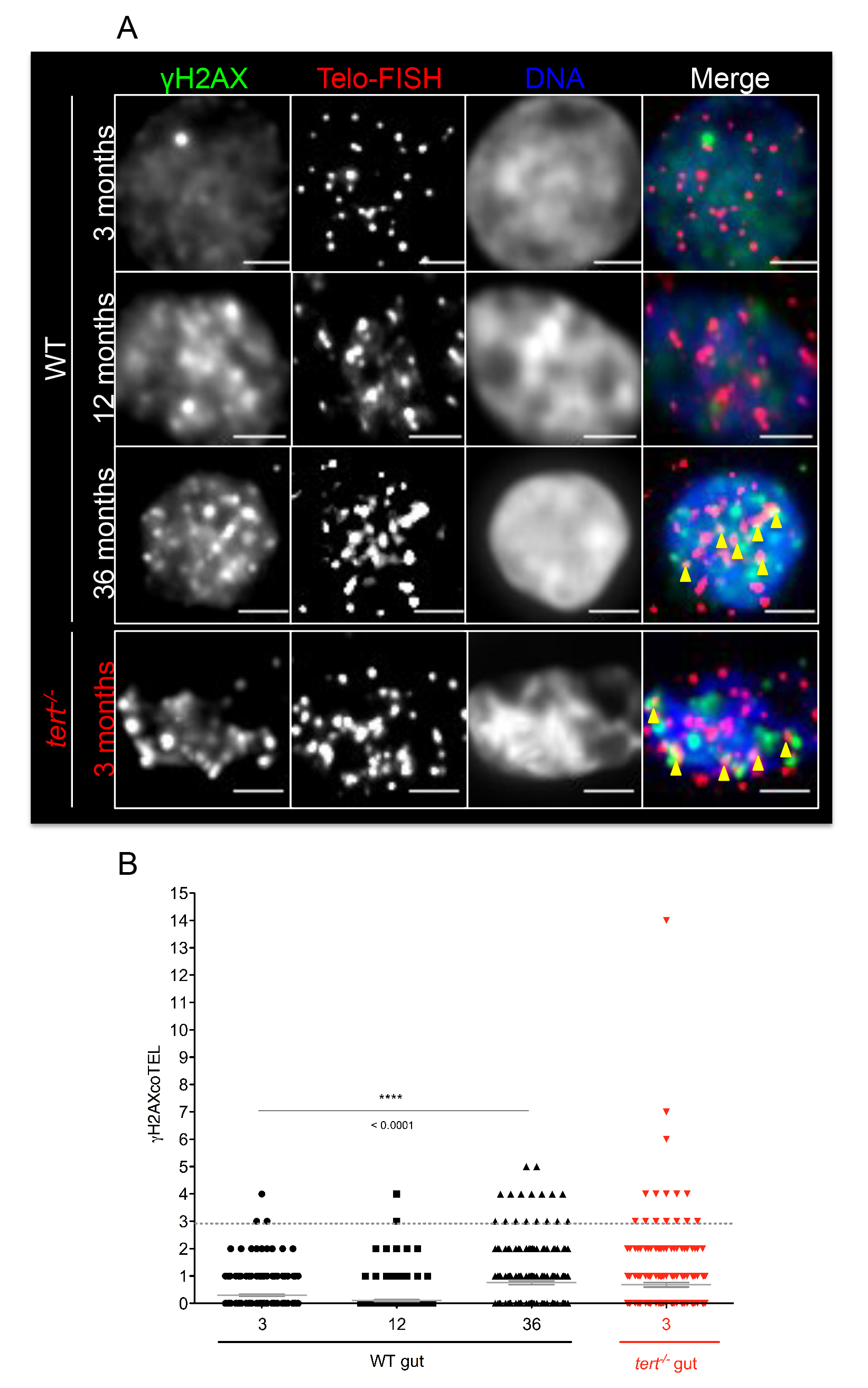

Supplement: S5 Fig — A) Representative immunofluorescence staining of γ-H2AX foci (green) and telomeres by FISH (red) in cells isolated (cytospin) from the gut of 3, 12 and 36 month-old WT and 3-months tert-/-. Yellow arrowheads point to co-localization of γ-H2AX and telomere signal–TIFs. Compared to 3-months, the number of TIFs increases significantly with age in the gut of 36 months (p<0.0001) and 3 month-old tert-/- zebrafish (p<0.0001), as shown by quantifications in B). Scale bar = 1 μm. Data are represented as mean +/- SEM. N = 3 individuals, per age per time point per genotype (N = 250 cells). (TIF) [file pgen.1005798.s005.tif]

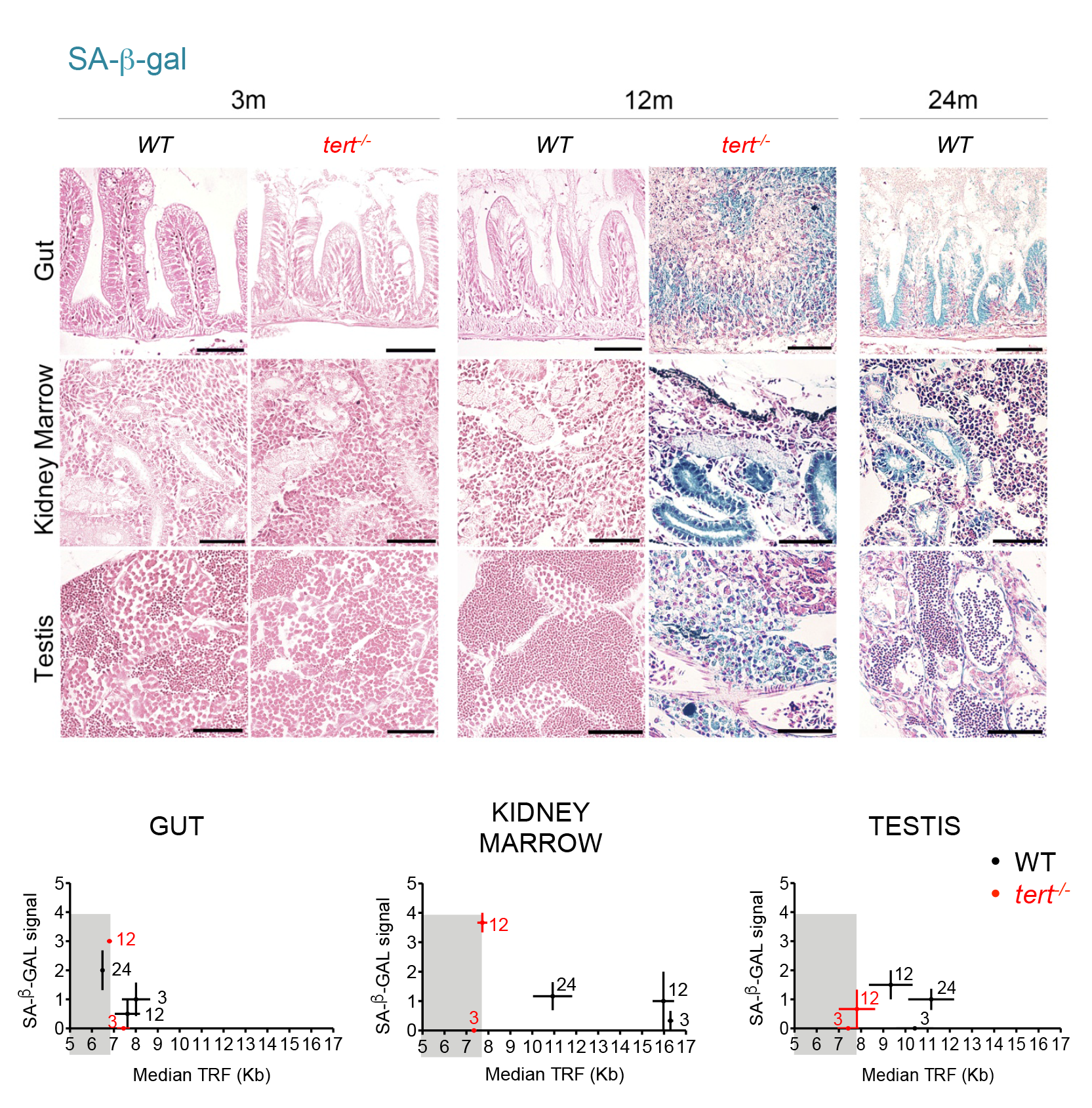

Supplement: S6 Fig — A) Representative haematoxylin-eosin and senescence-associated β-galactosidase (SA-β-gal) staining in gut, kidney marrow and testis sections of WT (at 3, 12 and 24 months) and tert-/- mutant siblings (at 3 and 12 months). A significant increase in SA-β-gal positive cells is seen by 12 months of age in tert-/- tissues and by 24 months in WT tissues (when comparing both with 3 month-old controls). No staining is observed in the muscle. B) Increase in SA-β-gal staining does not significantly correlate with mTL decline in any of the tissues tested. Grey shaded area identifies the telomeric length at which significant SA-β-gal staining is observed in tert-/- mutants’ tissues. WT and tert-/- age groups are indicated in each graph by black and red colored numbers, respectively. Quantifications of SA-β-GAL signal were performed using an arbitrary scale of signal intensity (where weaker signal is represented by 1 and stronger signal by 4). N = 3–6 per genotype per time point. Scale bar = 50 μm. N = 3–6 for tissue mTL telomere length quantifications per genotype per time point (x-axis in graphs of S6B Fig). Data are represented as mean +/- SEM. (TIF) [file pgen.1005798.s006.tif]

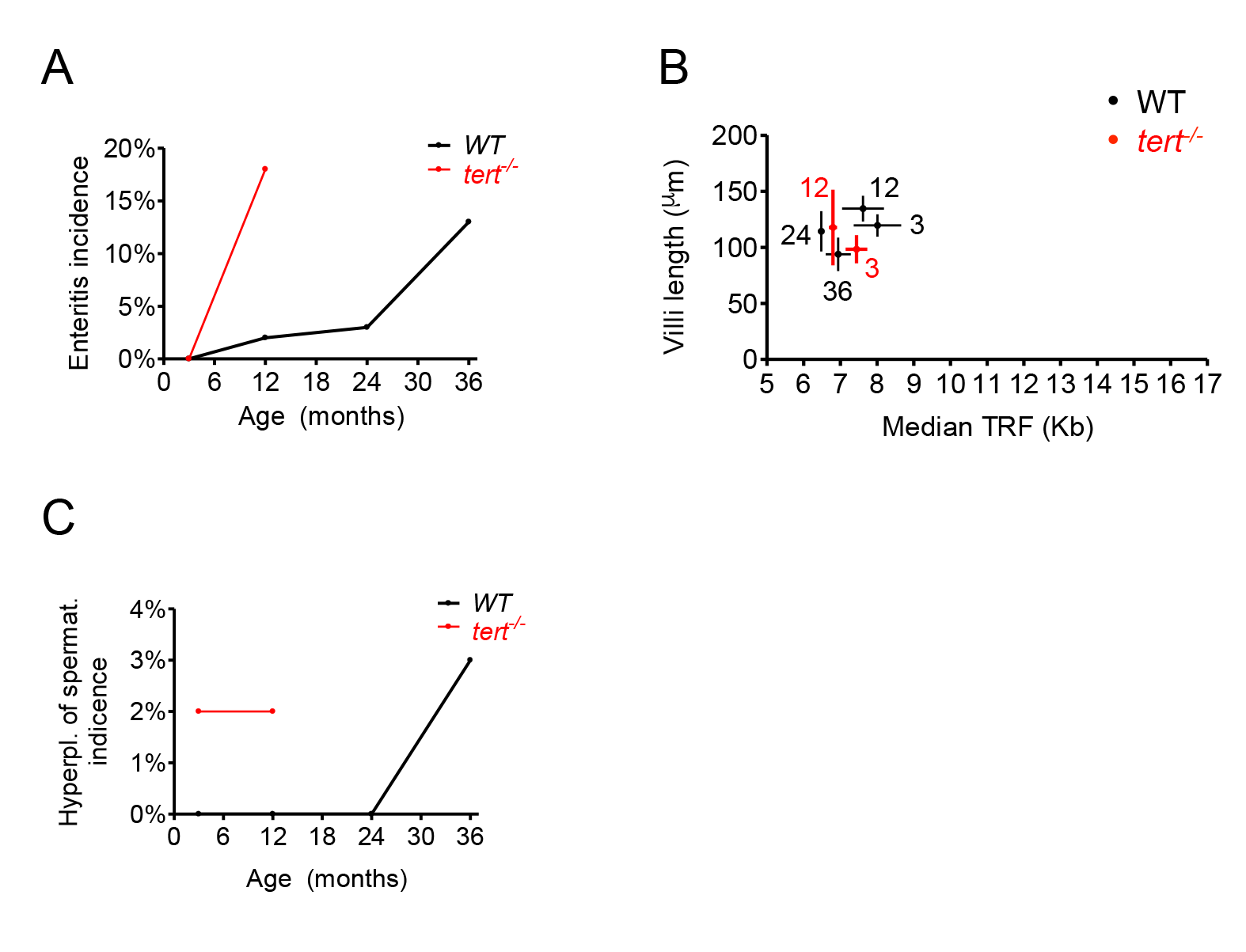

Supplement: S7 Fig — Quantification of the incidence of intestinal inflammatory cell infiltration (enteritis), gut villi length and hyperplasia of the testis in both WT and tert-/- mutants with aging. A) As WT and tert-/- mutants age, there is progressive inflammatory cell infiltration of the lamina propria in the gut (particularly after the age of 24 months in WT and from 6 months onwards in tert-/- mutants). WT show 13% of enteritis incidence by 36 months (N = 30/238) and tert-/- have 18% incidence by 12 months (N = 12/ 66). B) No differences in gut villi length are found during WT or tert-/- aging (quantification of 5 different fields of view for 4–5 different individuals per time point per genotype). WT and tert-/- age groups are indicated in each graph by black and red colored numbers, respectively. C) The percentage of zebrafish with hyperplasia of seminiferous epithelium raises to 3% in 36 months WT cohorts (N = 7/238), accompanied by a progressive decrease in fertilization capacity with age (Fig 4C), but maintains at very low levels in tert-/- mutants (N = 1/66). (TIF) [file pgen.1005798.s007.tif]

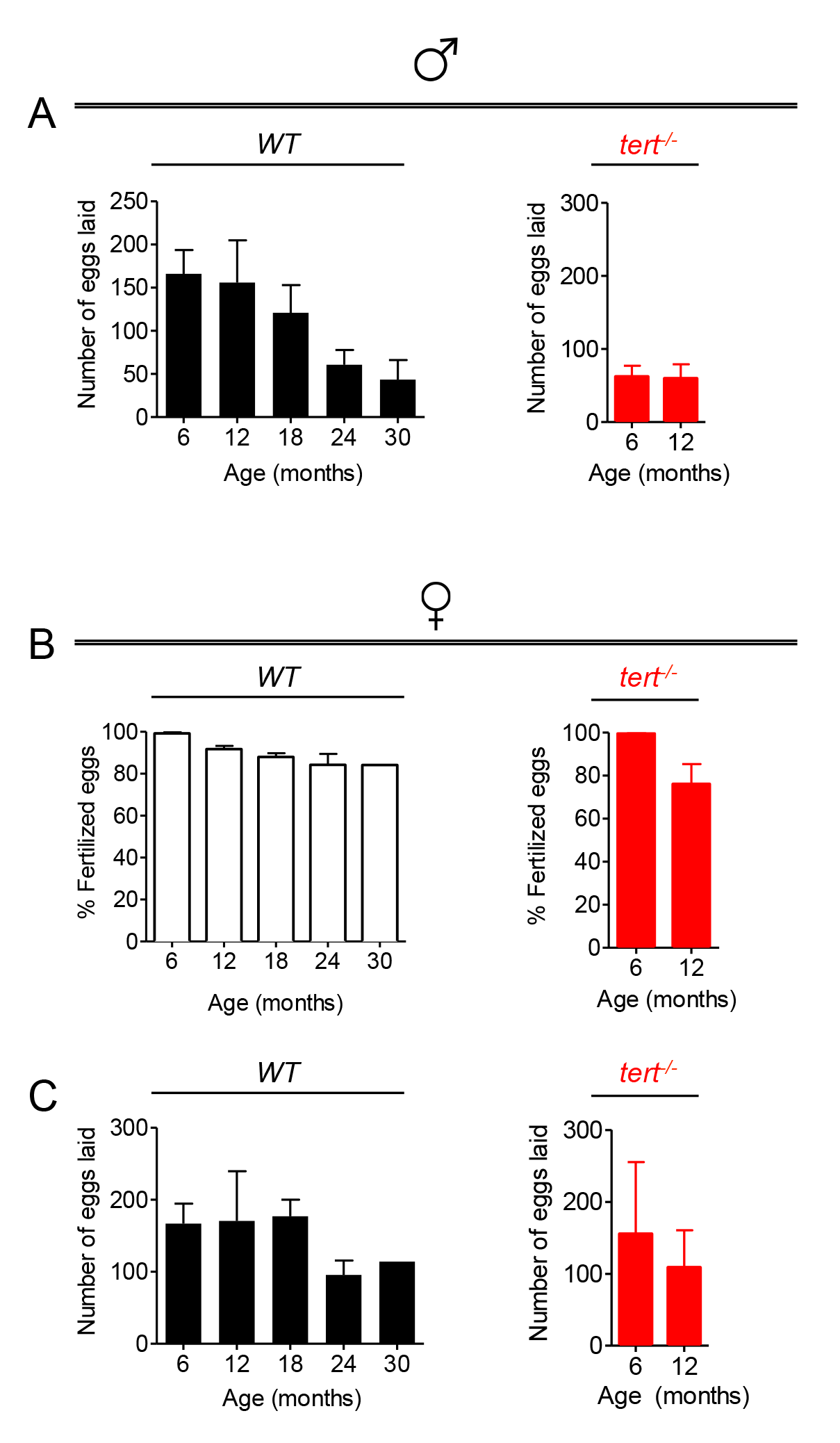

Supplement: S8 Fig — A) Fertility in WT male zebrafish declines by ca. 50% at 24 months (Fig 4C), and is accompanied by a reduced ability to stimulate female spawning (average of 61 vs. 166 eggs layed in crosses with 24 month-old vs. 3 month-old males, 3–10 fertilization trials per time point). tert-/- mutant males show defects in egg spawning stimulation already by 6 months (average of 62 vs. 166 eggs layed in crosses with 3 month-old tert-/- vs. 3 month-old WT males, 3–10 fertilization trials per time point). B) Conversely, mild reproductive function defects are seen in 24-month vs. 6-month old WT females (ca. 84% vs. 99% reproductive function, respectively, 3–10 fertilization trials per time point). tert-/- females show a slight defect (ca. 76%) in the percentage of fertilized eggs by 12 months of age when compared with 6 months (3 fertilization trials per time point). C) Female spawning is not significantly affected by age in WT (3–10 fertilization trials per time point) or tert-/- female populations (3 fertilization trials per time point). (TIF) [file pgen.1005798.s008.tif]

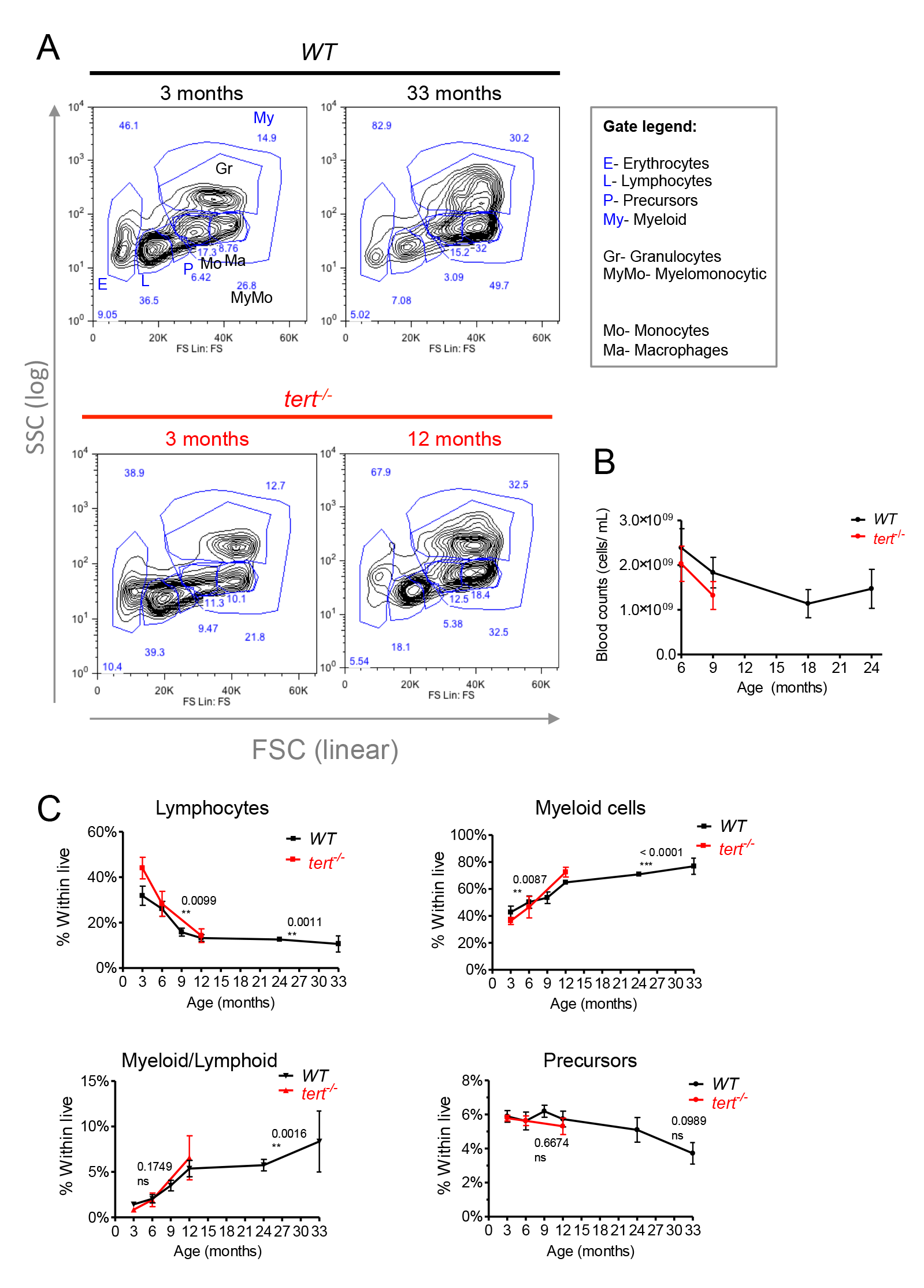

Supplement: S9 Fig — A) Identification of zebrafish kidney marrow cells by flow cytometry and B) quantification of the percentage of lymphocytes, myeloid cells, myeloid/lymphoid ratios and precursors for WT (3, 6, 9, 12, 24 and 33 months) and tert-/- mutants (3, 6, 9 and 12 months). B) The percentage of WT lymphocytes (“L” gate in A) declines from 30% at 3 months to 10% at 33 months (p = 0.0011). tert-/- mutants also show a decline from 3 to 12 months (p = 0.0099) which accompanies that of WT zebrafish for the same age interval. The percentage of WT myeloid cells (“My” gate in A) increases gradually from 2% at 3 months to 9% at 33 months (p<0.0001). tert-/- mutants’ percentage of myeloid cells follows the tendency of WT with age, gradually increasing from 3 to 12 months. Consequently, myeloid/lymphoid ratios increase with age in WT kidney marrow. No significant changes are found in WT and tert-/- populations of precursors (“P” gate in A) with age. N = 3–5 per time point per genotype. (TIF) [file pgen.1005798.s009.tif]

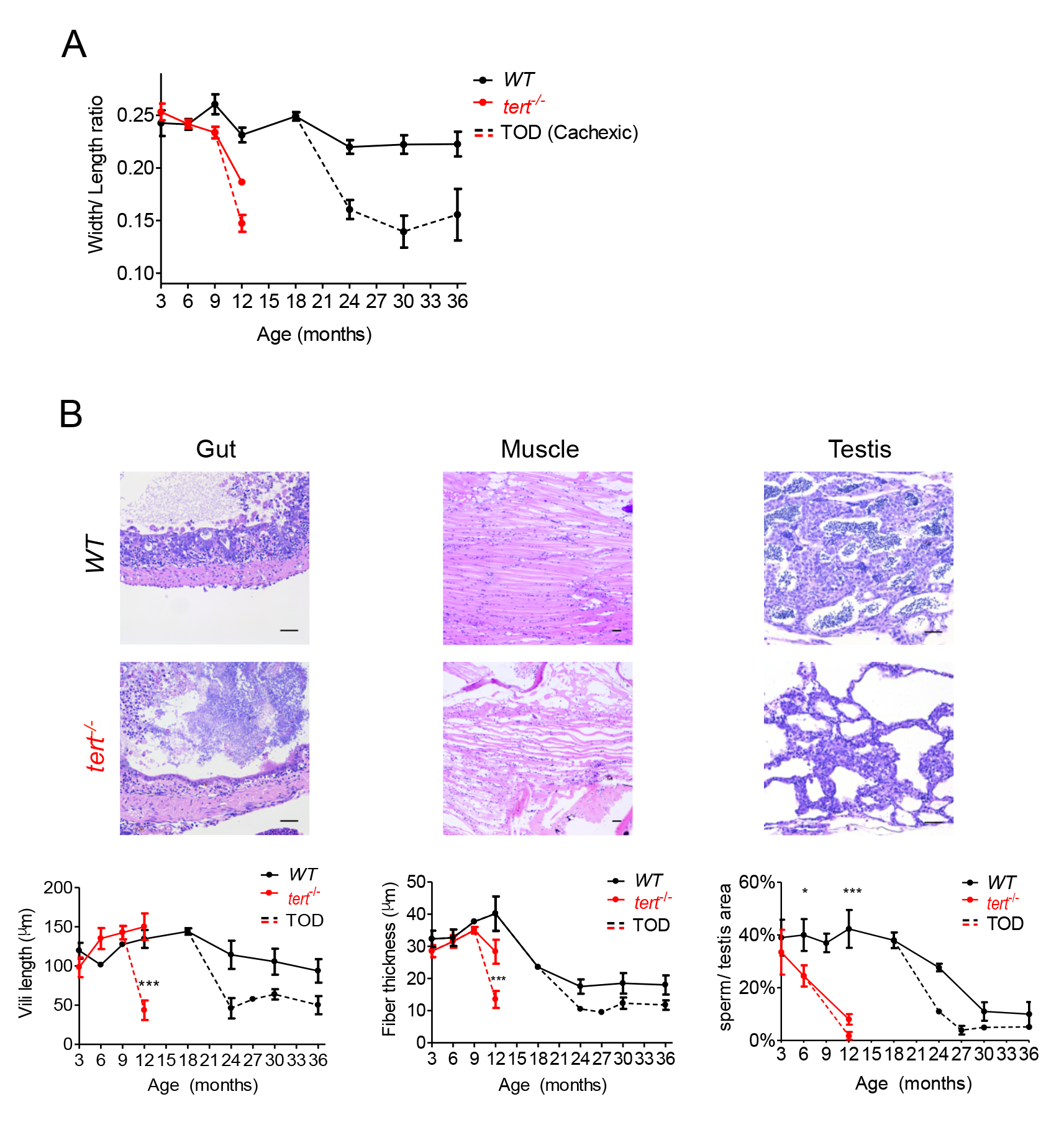

Supplement: S10 Fig — A) Quantification of width/length ratios shows that at time of death (TOD), both WT and tert-/- zebrafish have significantly lower body mass indexes when compared with non-cachexic siblings (N = 4–17 per time point for WT zebrafish and N = 3–7 for tert-/- mutants). B) Representative hematoxilin and eosin-stained sections of gut, muscle and testis from WT and tert-/- siblings at TOD. B) Cachexia is associated with shorter villi and/or villous atrophy (defined as flattening of the gut mucosal surface, N = 3–6 per genotype per time point) and severe myocyte atrophy and degeneration (N = 3–6 per genotype per time point), to a higher degree than that found for non-cachexic siblings. Testis also shows pronounced atrophy, with reduced germ cell compartment associated with cachexia (N = 3–4 per genotype per time point). TOD corresponds to the interval comprising the second and third quartiles of survival (25 to 75%). Quantifications were performed in at least 3 different fields of view for each individual. Scale bar = 50 μm. Data are represented as mean +/- SEM. (TIF) [file pgen.1005798.s010.tif]

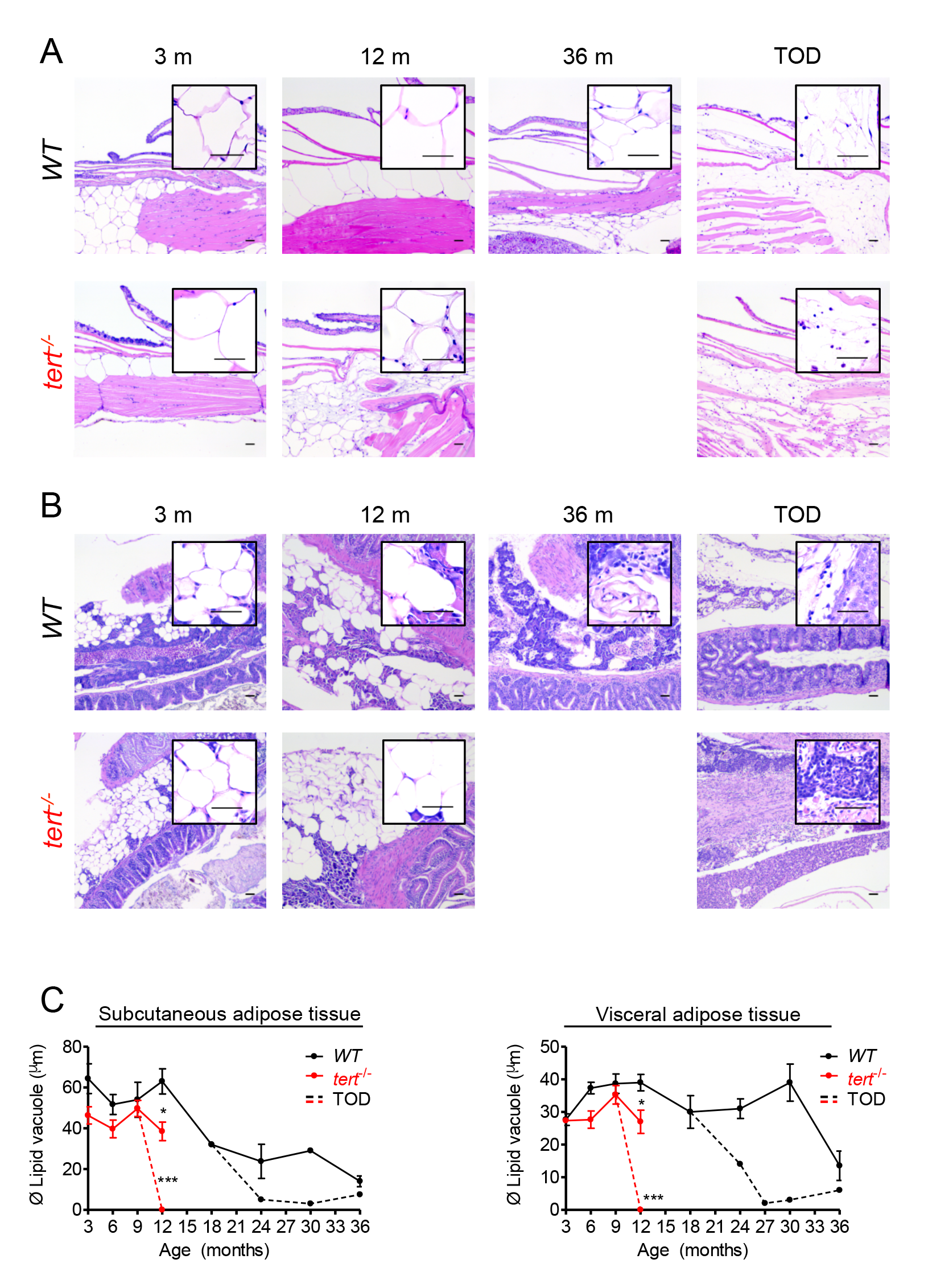

Supplement: S11 Fig — Representative hematoxilin and eosin-stained sections of subcutaneous (A) and visceral (B) adipose tissue depots of WT (3, 12 and 36 months and TOD) and tert-/- siblings (3 and 12 months and TOD), and C) quantification of the adipocyte vacuole diameter at different ages. A) WT zebrafish show a progressive loss of the subcutaneous depot, with age, C) accompanied by a reduction in the adipocytes’ vacuole diameter (adipocytes are ~3.3 times smaller at 36 months vs. 3 months). tert-/- anticipate this phenotype by 12 months of age. With cachexia, both WT and tert-/- zebrafish show complete exhaustion of the subcutaneous adipose tissue depot (at TOD). B) Visceral adipose tissue (peri-pancreatic) is reduced or absent at 36 months in WT zebrafish (when adipocytes are ~3 times smaller than at 3 months) and by 12 months in WT mutants; and similarly to subcutaneous depot, exhaustion of visceral adipose reserves associates with cachexia. N = 3–7 zebrafish per genotype per time point. Time of death (TOD) corresponds to the interval comprising the second and third quartiles of survival (25 to 75%) Scale bar = 50 μm. Data are represented as mean +/- SEM. (TIF) [file pgen.1005798.s011.tif]

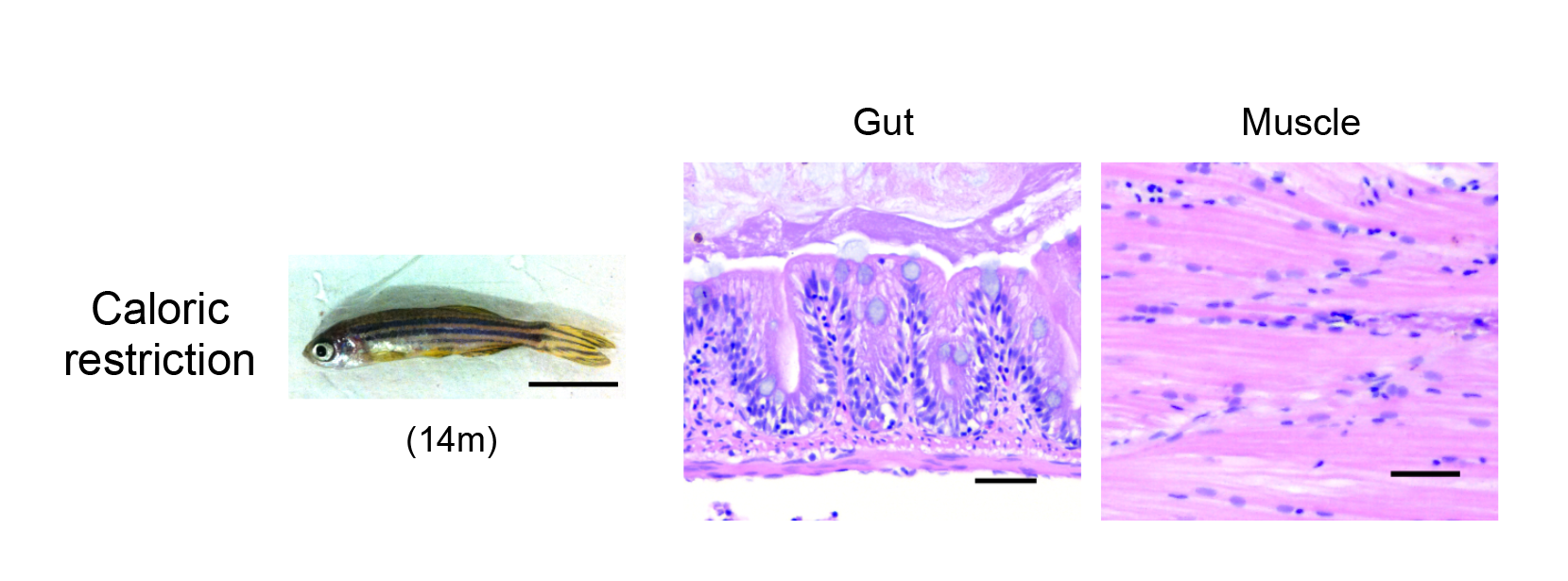

Supplement: S12 Fig — Representative picture and hematoxilin and eosin-stained sections of 12-month old WT after 8 weeks of caloric restriction, where food intake was reduced by 85%. Upon caloric restriction, gut villi length is maintained within a normal range (when compared with non cachexic 12-month old WT–see Fig 4A) but myocytes show atrophy to a similar degree as seen in cachexic WT and tert-/- zebrafish. N = 4. Scale bar = 50 μm. (TIF) [file pgen.1005798.s012.tif]

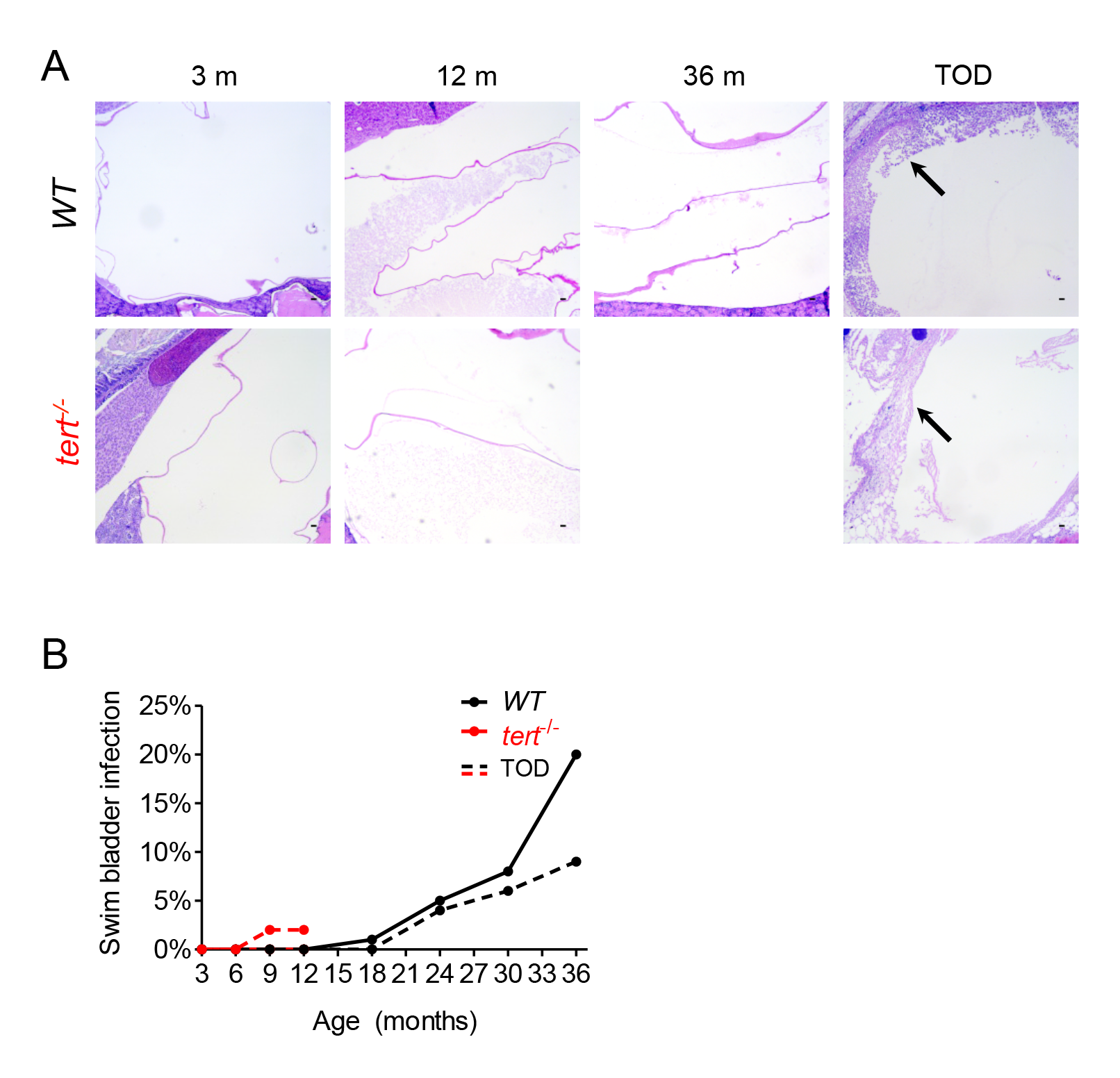

Supplement: S13 Fig — A) Representative hematoxilin and eosin-stained sections of swim bladder from WT (3, 12, 36 months and TOD) and tert-/- mutants (3, 12 months and TOD). There is massive destruction and inflammation of the swim bladder at time of death (indicated by black arrow), associated with cachexia, which is accompanied by necrosis and often extended throughout the visceral cavity, both in WT and tert-/- mutants, compatible with aerocystitis. B) Incidence rates reach 30% in 24 month WT zebrafish (N = 71/238) and 3% in 9-month old tert-/- mutants (N = 2/66). TOD corresponds to the interval comprising the second and third quartiles of survival (25 to 75%). Scale bar = 50 μm. Data are represented as mean +/- SEM. (TIF) [file pgen.1005798.s013.tif]
